# Long terminal repeats (LTR) and transcription factors regulate *PHRE1* and *PHRE2* activity in Moso bamboo under heat stress

**DOI:** 10.1186/s12870-021-03339-1

**Published:** 2021-12-09

**Authors:** Pradeep K. Papolu, Muthusamy Ramakrishnan, Qiang Wei, Kunnummal Kurungara Vinod, Long-Hai Zou, Kim Yrjala, Ruslan Kalendar, Mingbing Zhou

**Affiliations:** 1 State Key Laboratory of Subtropical Silviculture, Zhejiang A&F University, Lin’an, Hangzhou, 311300 Zhejiang China; 2 Co-Innovation Center for Sustainable Forestry in Southern China, Nanjing Forestry University, Nanjing, 210037 Jiangsu China; 3 Bamboo Research Institute, Nanjing Forestry University, Nanjing, 210037, Jiangsu China; 4 Division of Genetics, ICAR - Indian Agricultural Research Institute, New Delhi, 110012 India; 5 Helsinki Institute of Life Science HiLIFE, Biocenter 3, Viikinkaari 1, FI-00014 University of Helsinki, Helsinki, Finland; 6 Zhejiang Provincial Collaborative Innovation Centre for Bamboo Resources and High-efficiency Utilization, Zhejiang A&F University, Hangzhou, 311300 Zhejiang China

**Keywords:** Moso bamboo, LTR-retrotransposons, Transposable elements, *PHRE1* and *PHRE2*, siRNA, Heat stress, Transposition

## Abstract

**Background:**

LTR retrotransposons play a significant role in plant growth, genome evolution, and environmental stress response, but their regulatory response to heat stress remains unclear. We have investigated the activities of two LTR retrotransposons, *PHRE1* and *PHRE2*, of moso bamboo (*Phyllostachys edulis*) in response to heat stress.

**Results:**

The differential overexpression of *PHRE1* and *PHRE2* with or without CaMV35s promoter showed enhanced expression under heat stress in transgenic plants. The transcriptional activity studies showed an increase in transposition activity and copy number among moso bamboo wild type and *Arabidopsis* transgenic plants under heat stress. Comparison of promoter activity in transgenic plants indicated that 5’LTR promoter activity was higher than CaMV35s promoter. Additionally, yeast one-hybrid (Y1H) system and *in planta* biomolecular fluorescence complementation (BiFC) assay revealed interactions of heat-dependent transcription factors (TFs) with 5’LTR sequence and direct interactions of TFs with *pol* and *gag*.

**Conclusions:**

Our results conclude that the 5’LTR acts as a promoter and could regulate the LTR retrotransposons in moso bamboo under heat stress.

**Supplementary Information:**

The online version contains supplementary material available at 10.1186/s12870-021-03339-1.

## Background

Abundantly present in plant genomes, long terminal repeat (LTR) retrotransposons, class I transposable elements (TE), are characterized by a pair of identical repeats several hundred base pairs long on both the terminals. They belong to two major superfamilies, Ty1/*Copia*, and Ty3/*Gypsy* with pan-genome distribution and constitute a major portion of genomes [1, 2]. The most common internal coding genes flanked by the LTRs are *gag* (group-associated antigene or group-specific antigen) and *pol* (polymerase), involved in RNA synthesis. The *pol* is involved in reverse transcription and encodes pepsin-like aspartate proteases (PR), integrase (INT), reverse transcriptase (RT), and ribonuclease H (RH) proteins. The *gag* is involved in the maturation, packaging of RNA-mediated RTs, and re-integration into the genome with the help of INT. LTR retrotransposons have other structural features such as primer binding site (PBS) and a poly-purine tract (PPT), necessary for reverse transcription and transposition [3, 4]. The transcriptional activity of LTR retrotransposons is regulated by promoter elements in the 5’ LTR and untranslated (UTR) regions. Due to their ‘copy and paste’ transposition activity LTR retrotransposons can generate new mutations within the genome [4, 5], including copy insertion, gene silencing, chromosomal rearrangements, and genome amplification. Although LTR retrotransposon mutations occur at random in an organism, only the most beneficial mutations are carried forward [6, 7].

Natural triggers of transposition activity of LTR retrotransposons remain unclear, but it is well documented that they can be activated by stress instances [8], and bear some epigenetic marks. In tobacco, the specific expression of the retroelements, *Tnt1* and *Tto1* situated within the U3 region of 5’LTR fused by the *GUS* reporter gene have revealed transcriptional activity under the stress conditions [9–11]. Integrated into the genome of *Arabidopsis* and rice, *Tnt1* and *Tto1* attained transposition under stress conditions [12, 13]. Similarly, in oat (*Avena sativa*), a Ty1/*Copia* retrotransposon *OARE1* was detected highly expressed under biotic and abiotic stresses [14], thus stimulating plant defense responses. A retrotransposon in *Citrus limon*, *CLCoy1* was induced by salt and wounding stresses [15]. A recent study suggests that heat-activated *Copia* superfamily LTR retrotransposons in *Arabidopsis* increased nuclear size and strengthened chromatin reorganization [16]. Compared to normal conditions, GBRE-1 elements in *Gossypium barbadense* and *G. hirsutum* showed higher expression levels under heat stress [17]. *HuTy1P4* retroelement in the pitaya (*Hylocereus undatus*) genome is transcriptionally activated by different stress conditions [18]. *ONSEN*, an element of Ty1/*Copia* superfamily, in *Arabidopsis* progeny lines was found to be most effective under heat stress [19].

Despite being significant components of the plant genome, most LTR retrotransposons remain silent because of the suppression by DNA and histone proteins modifications, recombination, and small RNAs [20–22]. With the presence of trans-acting small interfering RNAs (siRNAs), usually produced in high copy numbers, LTR retrotransposons are involved in gene regulation at the transcriptional and post-transcriptional levels [20, 23–25]. Heat stress adaptation in *Arabidopsis* is reported to activate a Ty1/*Copia* retrotransposon named *ONSEN*, which was found activated in mutants impaired in the biogenesis of siRNAs [26, 27]. The transcriptional activation of *ONSEN* was regulated by the siRNA-related pathway and showed transgenerational transposition of the retroelement under heat stress [28]. Evidence for post-transcriptional and epigenetic control of *Gypsy* retroelements through regulation of piwi-interacting RNA (piRNA) have been reported in *Drosophila*, in which a heat-responsive stress chaperone heat shock protein 70 (HSP70) was found inducing the transposon activity [29]. The experience from plants so far indicates that LTR retrotransposons functions are species-specific and are associated with environmental adaptation and provides an evolutionary advantage [30–32].

Among the bamboos, moso bamboo, *Phyllostachys edulis* (subfamily Bambusoideae) stands prominent among the 500 species belonging to 48 genera [33, 34] and is recognized for its economic use. It is acclaimed as one of the fastest-growing plants on earth, with a growth rate of 30 to 100 cm per day [35, 36]. Although grown in a wide range of climates, moso bamboo is typically temperate adapted and shows a long vegetative phase (usually 60 years) and monocarpic. The switch from a vegetative to a reproductive phase is unpredictable [37, 38]. Controlled cross-breeding and development of breeding lines and mapping populations are difficult in moso bamboo, and therefore, in genetic studies, it lags far behind other cultivated cereals [36]. The moso bamboo is large with a size closer to 2.0 Gbp but is smaller than the maize genome [39]. Transposons occupy over 63.24% of the moso bamboo genome [40] consisting of 45.67% of retroelements. Among the retroelements, LTR retrotransposons occupy about 878 Mbp (43.89%), a size equivalent to 2.25 times that of the rice genome [41–46].

In our previous studies, we have reported two LTR retrotransposons *PHRE1* and *PHRE2* (synonymized with *ph*-LTR1 and *ph*-LTR2, respectively) in the moso bamboo genome [47, 48]. Selected based on the homology and structure with that of LTR retrotransposons, *PHRE1* and *PHRE2* contain the RT, RH, INT genes, PBS, and PPT, qualifying them to be capable of transposition. The open reading frames of these protein domains were complete and had no distinct mutations. The terminal repeats of 5′ and 3′ sequences of *PHRE1* (98.5%) and *PHRE2* (98.3%) share significant sequence homology. Zhou et al. [47, 48] found that under irradiation treatment, *PHRE2* copy number increased in moso bamboo seedlings as well as in transgenic *Arabidopsis* plants. In *Arabidopsis*, however, higher transposition activity could be noticed T3 plants than in the T2 plants, and a detailed molecular and functional characterization has not been attempted in these studies [47, 48], analyzing the information on the native retrotransposon functionality. Thus, we have carried out this study to characterize molecular functions of *PHRE1* and *PHRE2* to fathom the basic retrotransposon functions as well as the promotor-mediated epigenetic regulation in response to heat stress. For characterization, we used the modified carbon nanotube diffusion method for moso bamboo transformation [49] and *Agrobacterium* floral dip method for *Arabidopsis* transformation to overexpress these elements.

## Results

### Structure analysis of *PHRE1* and *PHRE2*

*PHRE1* and *PHRE2* were selected based on the complete structure and homology of domains, complete with two full-length *gag* and *pol* genes with continuous ORFs without nonsense and frameshift mutations (Table S1). Both were Ty3/*Gypsy* LTR retrotransposons with 4980 bp (*PHRE1*) and 5515 bp (*PHRE2*) length [47, 48]. The 5’LTR of *PHRE1* contained core promoter features such as four CAAT boxes located at 39, 142, 144, and 291 bp positions and TATA box located at 49 bp positions. Two methyl jasmonate regulatory elements of TGACG-motif were located at 20 and 311 bp positions, and a GTGGC-motif was located at 10 bp position. Three drought-responsive myeloblastosis (MYB) binding sites (MBS) were located at 16, 104, and 219 bp positions, and a light-responsive MYB-recognition element (MRE) site is located at 215 bp positions. In the *PHRE2* element, the 5’ LTR (670 bp) and 3’LTR (465 bp) of shared significant homology sequences (98.3%). The core promoters, TATA box, and CAAT box were located at 280 and 267 bp positions, respectively. Additionally, *PHRE2* had a salicylic acid (SA) related element at 193 bp, gibberellin (GA) related element at 94 bp, and temperature-responsive elements at 212 bp and 133 bp positions. The promoter also had *cis*-regulatory elements, such as methyl jasmonic acid (CCTGCA), auxin regulatory (TGA), drought-responsive (MYB), and abscisic acid (ABA) regulatory elements located at 193, 94, 212, and 133 bp respectively [47, 48].

### Development of transgenic plants

At least 40 *Arabidopsis* primary transformants (T0) of each *PHRE1* and *PHRE2* were generated using floral dip transformation. After hygromycin selection, the plants were established in a growth chamber where they developed normally and set seeds. Screening of putative transformants in T1 generation in the presence of 30mg/L hygromycin resulted in survival (exhibited proper flowering, shoot, and root formation) of at least 20–25 independent events for each *PHRE1* and *PHRE2* (Fig S1). After 45 days, T1 plants were phenotypically and morphologically (flowering, shoot, root, plant height, and seed setting) similar to untransformed control plants grown under a non-selective medium. This indicated that neither the antibiotic resistance gene nor the LTR retrotransposons constructs had affected the growth of transformed plants. PCR analysis using primers specific for *PHRE1*, *PHRE2*, *GUS*, and antibiotic genes, confirmed the presence of T-DNA in the putative transformants (Fig. S2). Likewise, at least 20 moso bamboo transgenic plants were developed using carbon nanotubes transformation after 3 days post-infiltration. Reporter gene (*GFP*) expression was observed in moso bamboo transgenic plants by confocal microscopy imaging and performed qRT-PCR assay for the transformants.

### *PHRE1* and *PHRE2* show promoter activity in moso bamboo

**Fig. 1:**
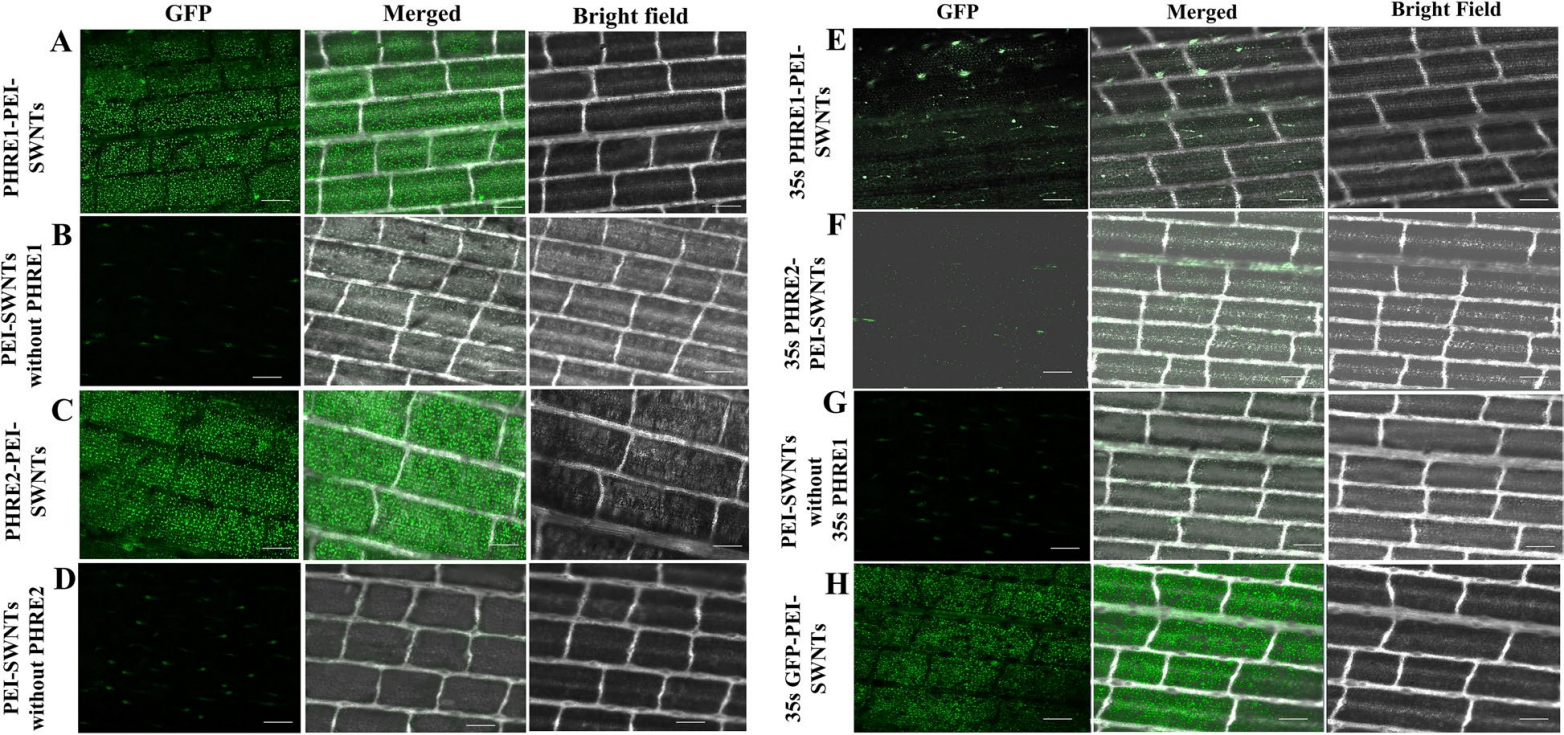
Confocal microphotographs showing *GFP* expression of *PHRE1* and *PHRE2* transformed into mature leaves of moso bamboo using the carbon nanotube diffusion method (PEI-SWNTs). **A**, **C** The *GFP* expression of *PHRE1* and *PHRE2* plasmid DNA-PEI-SWNTs trafficking in moso bamboo plant cells, **B**, **D** Carbon nanoparticle internalization into mature leaf cells shown by imaging PEI-SWNTs without plasmid DNA (pMDC164, 5’LTR promoter). **E**, **F** The *GFP* expression of *PHRE1* and *PHRE2* plasmid DNA-PEI-SWNTs (pMDC43 with 35s promoter) trafficking in moso bamboo plant cells. **G** PEI-SWNTs without plasmid DNA (pMDC43 with 35s promoter). **H** Carbon nanoparticle internalization into mature leaf cells shows by imaging PEI-SWNTs with plasmid DNA (35s-*GFP* as positive control). Scale bar represents 100 μm. LTR retrotransposons, long terminal repeat-retrotransposons; PEI-SWNTs, polyethyleneimine single-walled carbon nanotubes

**Fig. 2:**
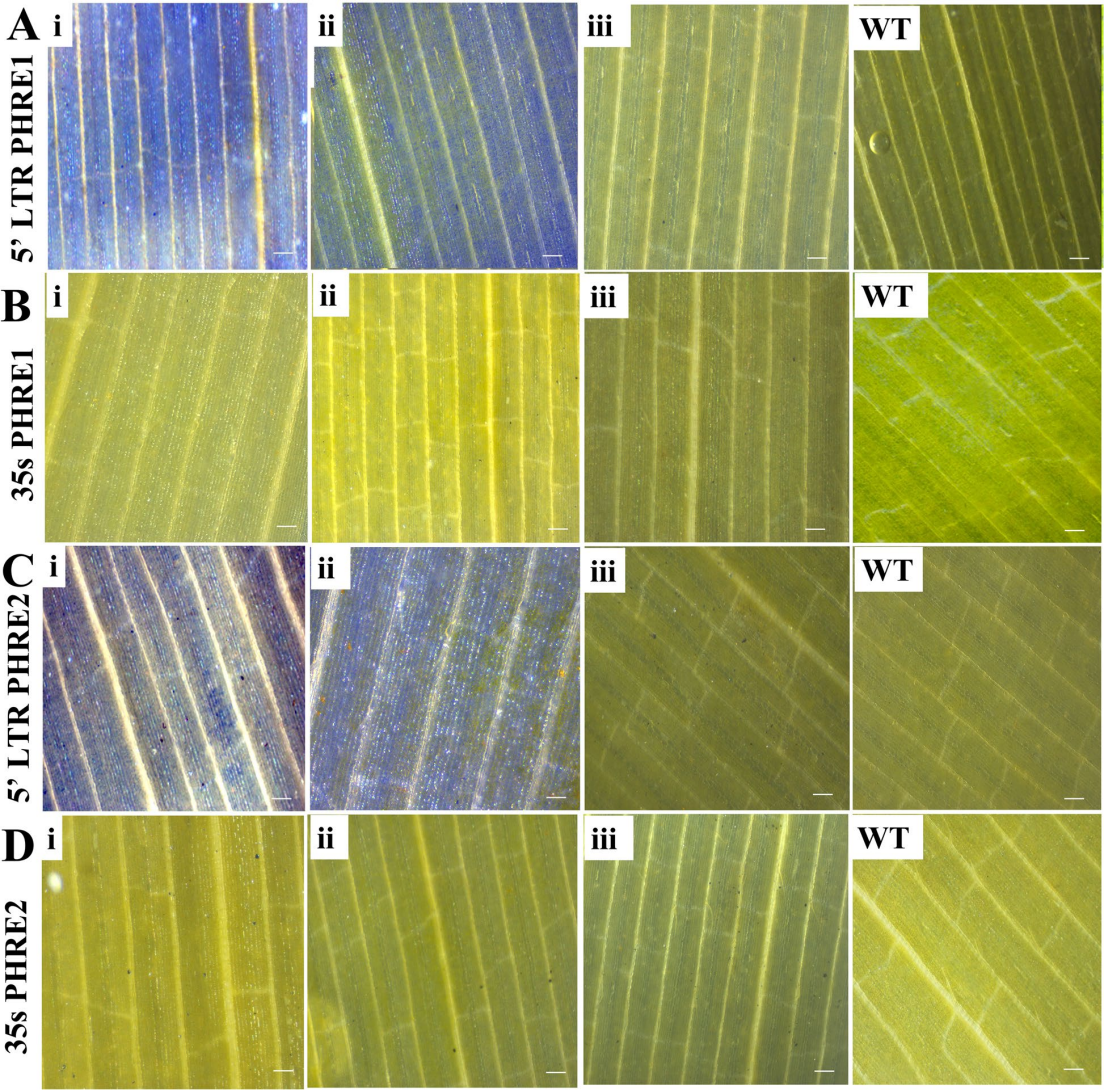
*GUS* expression of *PHRE1*:pMDC164, *PHRE2*:pMDC164, *PHRE1*:pMDC43, and *PHRE2*:pMDC43 in transgenic moso bamboo leaves using carbon nanotubes diffusion transformation. Photographs showing the stained mature and young leaves infiltered by (**A**, **C**-i-ii) *PHRE1* and *PHRE2* (pMDC164) and (**B**, **D**-i-ii) *PHRE1* and *PHRE2* (pMDC43). A, B, C, D – iii, and WT are PEI-SWNTs without plasmid DNA, and (WT) plasmid DNA, respectively. Scale bar represents 50 μm

**Fig. 3:**
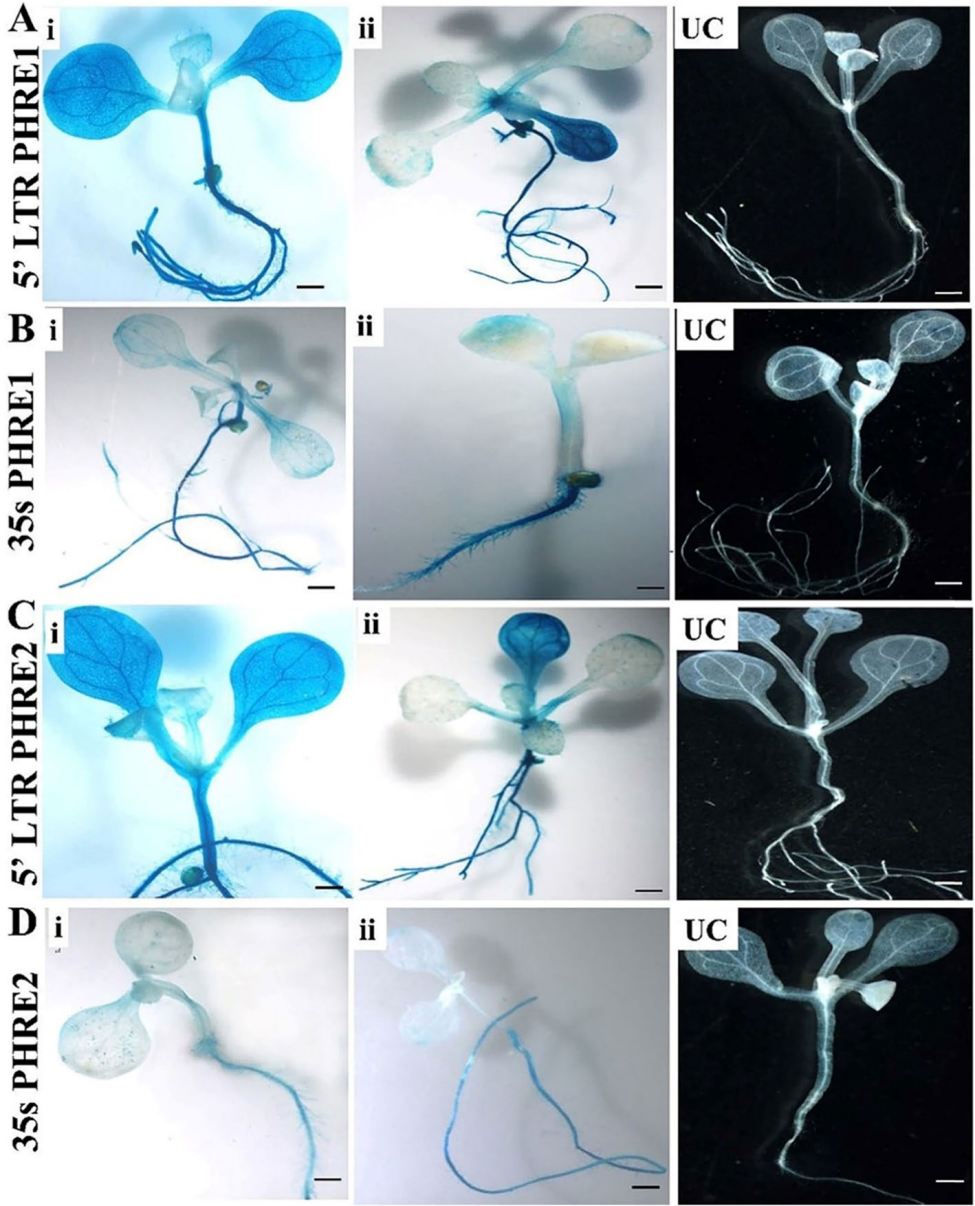
Transgenic *Arabidopsis* plants showing *GUS* expression analysis of *PHRE1* and *PHRE2*. **A**, **C** *GUS* expression analysis of pMDC164 construct of *PHRE1* and *PHRE2* in transgenic *Arabidopsis* plants. Four weeks old T1 seedlings were incubated at 37 °C for 24h. (i) T1 transformants harboring pMDC164 construct with *PHRE1* and *PHRE2* (ii) transgenic plant under normal conditions and UC. **B**, **D** (i) *GUS* histochemical staining of T1 transformants harboring pMDC43 construct with *PHRE1* and *PHRE2*. Four weeks old T1 seedlings were incubated at 37 °C for 24h, (ii) transgenic plants without stress, and UC. The blue color indicates the level of expression and the pMDC164 construct with *PHRE1* and *PHRE2* showed stronger expression than the pMDC43 construct with *PHRE1* and *PHRE2*. UC, untransformed control plant. Scale bar represents 20 μm

Observed under a confocal microscope, after the *GUS* assay incubation for 72h, the mature leaves of the transgenic moso bamboo plants indicated differential expression for the presence of promoters (Fig. 1 and 2). While no *GFP* and *GUS* expressions were observed in the system driven by the CaMV35s promoter, the systems are driven by *PHRE1* and *PHRE2* showed clear expression patterns with bright green fluorescence with GPF and blue color of *GUS* in the leaves (Fig.1 and 2). No fluorescent expression was detected in negative controls, including delivery of free plasmid DNA, DNA-PEI without SWNTs, and PEI-SWNTs without plasmid DNA (Fig.1). This indicated that the transcriptions of these reporter genes were driven by the promoter of *PHRE1* and *PHRE2*. In the transgenic *Arabidopsis* too, intense *GUS* staining was observed in the plants expressing the pMDC164:*PHRE1* and pMDC164:*PHRE2* (Fig. 3A and C) than pMDC43:*PHRE1* and, pMDC43:*PHRE2* (Fig. 3B and D). Notably, *GUS* transgene expression was absent in untransformed control plants. This implied that both *PHRE1* and *PHRE2* are active elements and have promoter activity.

The qRT-PCR on the mRNA from transgenic bamboo plants indicated that expression levels of 5’ and 3’ LTRs of *PHRE1* were down-regulated by 2.56- and 2.29- fold in the leaf, respectively, followed by the downregulation of *gag* and *pol* genes (1.93- and 1.90- fold changes) (Fig. S3 A). Similarly, 5’ and 3’ LTRs expression were down-regulated by 2.44- and 2.39- folds in the leaf of *PHRE2*, respectively, followed by the downregulation of *gag* and *pol* transcripts (1.95- and 1.96-fold changes) (Fig. S3 A). A similar expression was not observed in the transgenic bamboo with pMDC43:*PHRE1*/*PHRE2* having CaMV35s promoter. (Fig. S3 B). Also, in *Arabidopsis*, no transcripts corresponding to either *PHRE1* or *PHRE2* were detected in untransformed control plants. The gene expression data for the transgenic lines were presented relative to the *Arabidopsis actin* normalizer gene, and greater ΔCt values were obtained (difference between Ct mean of *PHRE1*/*PHRE2* and *Actin*) in *PHRE1* and *PHRE2* lines with CaMV35s promoter, than for *PHRE1* and *PHRE2* (without CaMV35s promoter). This indicated a lower quantitative expression of 5’LTR, *gag*, *pol*, and 3’LTR in the root, leaf, and stem of *PHRE1* and *PHRE2* with CaMV35s promoter (Fig. S4 and S5).

### *PHRE1* and *PHRE2* transcripts are expressed in roots and leaves

**Fig. 4:**
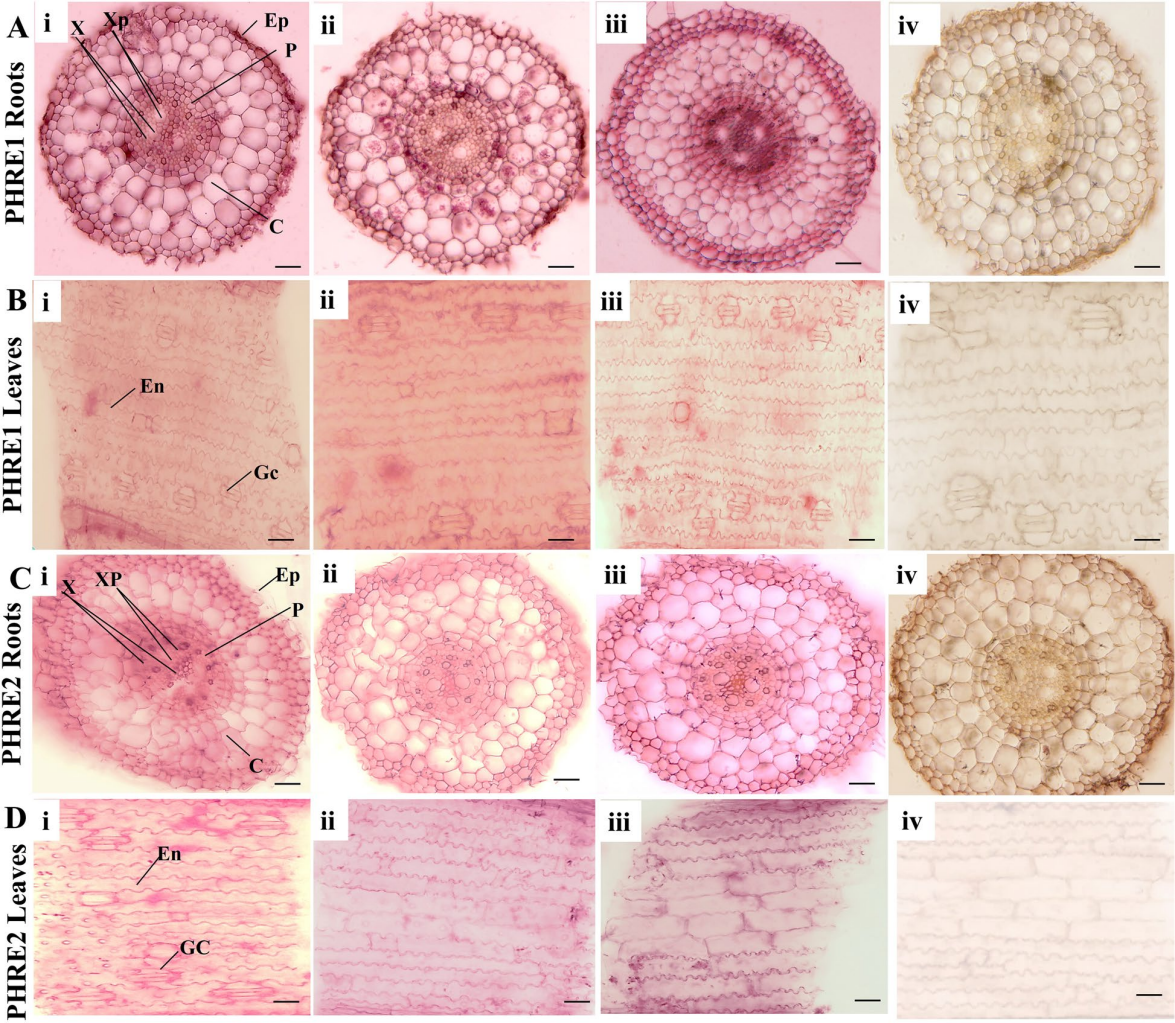
*In situ* hybridization assay shows the expression patterns of *PHRE1* and *PHER2* in moso bamboo roots and leaves. Location of *PHRE1* expression signified by purple/blue color due to the enzymatic cleavage of a chromogenic substrate (5- Bromo-4-chloro-3-indolyl phosphate/nitro blue tetrazolium) by alkaline phosphatase-conjugated to anti-DIG antibody. (A, C-i-iii) *In situ* hybridization of cross-sections of primary and lateral roots, roots, and (B, D-i-iii) mature leaf blades with *PHRE1* and *PHRE2* antisense probes. (A, B, C, D-iv) negative control with sense probes. Roots and leaves were sampled from 30-d-old seedlings treated under heat stress at 45 ^o^C for 4h. C, cortex; Ep, epidermis; P, pericycle; X, xylem; XP, xylem parenchyma; En, Endodermis and Gc, Guard cells. Scale bar represents 50 μm

The *in-situ* localization of *PHRE1* and *PHRE2* expression was identified in the root and leaves of bamboo. The probe hybridization using 306 bp and 301 bp fragments of 5’LTRs of *PHRE1* and *PHRE2*, respectively, showed significant expression patterns in root cortex (C), epidermis (Ep), pericycle (P), xylem (X), and xylem parenchyma (XP) whereas in the leaves it was detected in endodermis and guard cells. Similarly, the same expression of *PHRE2* was observed in the roots and leaves. Comparatively, *PHRE1* displayed more diffused staining that appeared to be localized in the cells associated with root cortical cells (Fig. 4A) whereas *PHRE2* expression was higher in the endodermis of leaf cells (Fig. 4D). These results were consistent with the RT-qPCR data. The hybridization signal was not detected in the roots and leaves using DIG-labeled sense probes of *PHRE1* and *PHRE2* (Fig. 4).

### *PHRE1* and *PHRE2* are activated by heat stress

**Fig. 5:**
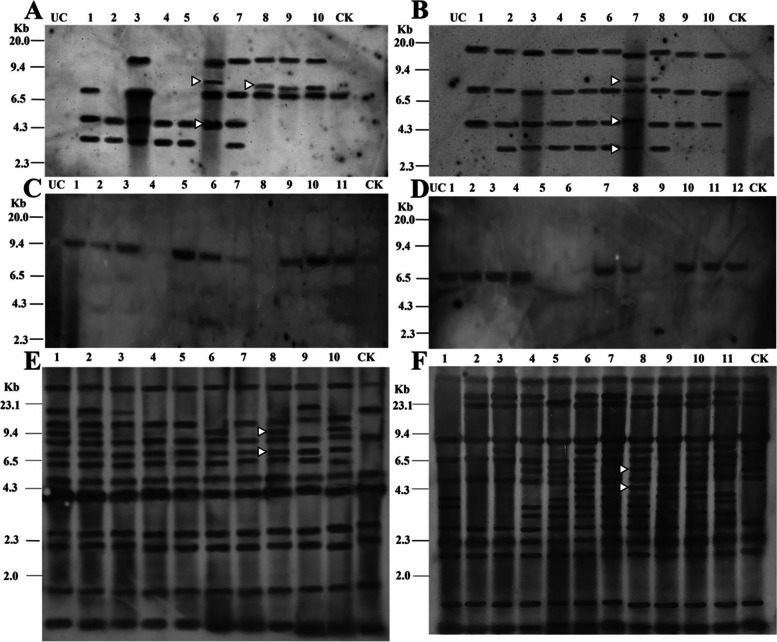
Southern hybridization of *Arabidopsis* T1 lines harboring the pMDC164 and pMDC43 constructs and moso bamboo wild-type plants. Genomic DNA isolated from untransformed/empty vector control (UC) plants did not show any hybridization signal. Stressed plants and non-stressed control (CK) plants, digested with Pac1 or HindIII enzyme showing hybridization signals, (A pMDC164, C pMDC43) *PHRE1* and (B pMDC164, D pMDC43) PHRE2 5’ LTR sequences were used as probes for hybridization of specific blots. Different lane numbers represent different transformant plants. Arrowheads indicate the transposed copies of LTRs. E and F, Southern hybridization for moso bamboo wild-type plants under heat stress. Genomic DNA isolated from stressed plants and non-stressed control (CK) plants, digested with *Hind*III enzyme showing hybridization signals. (E) *PHRE1* and (F) *PHRE2* LTRs were used as probes for the hybridization of specific blots. Different lanes represent different plants

Based on the reporter genes expression, we performed additional molecular analysis to identify the precise function of both elements. Eleven *Arabidopsis* T1 lines were subjected to Southern blot assay to analyze the insertion polymorphism of *PHRE1* and *PHRE2* under heat stress. The DNA blots were probed with 5’LTR sequences of *PHRE1* and *PHRE2* separately, and the transposition of *PHRE1* and *PHRE2* was observed in heat-stressed progeny lines driven by 5’ LTR promoter (Fig. 5A and B). *PHRE1* allowed better discrimination of T1 lines (1 to 10) of *Arabidopsis*, compared to the control plant. Similarly, transposition copies of *PHRE2* were observed in T1 lines 1 to 10. We did not observe any transposition of *PHRE1* and *PHRE2* among the transgenes driven by the CaMV35s promoter in control plants (Fig. 5C and D). This indicated that not only the 5’LTR acted as a promoter for stable integration, but also could inherit the *PHRE1* and *PHRE2* elements into the progeny plants. By Southern hybridization, similar transposition activity of *PHRE1* and *PHRE2* was observed in moso bamboo wild type plants exposed to heat stress as well (Fig. 5E and F). However, we did not use bamboo transgenic plants developed using the carbon nanotube diffusion method, for copy number detection since plasmid vectors could not integrate into the genome [49, 50].

**Fig. 6:**
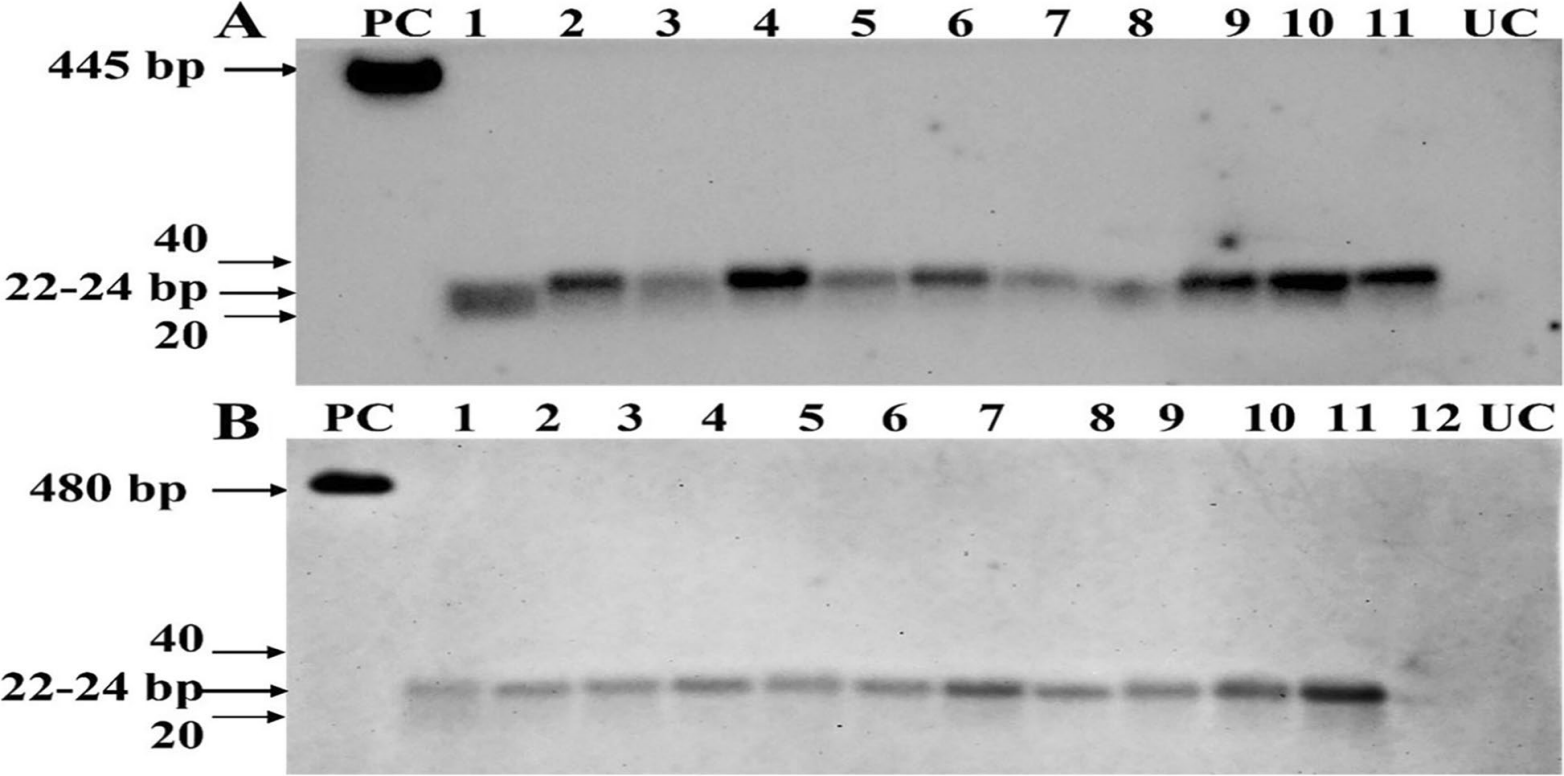
Northern blot analysis showing siRNA expression patterns of *PHRE1* and *PHRE2* LTR in *Arabidopsis* T1 lines. siRNA expression in the T1 progeny plants containing (A) *PHRE1* and (B) *PHRE2*. siRNA isolated from untransformed control (UC) plants did not show any hybridization signal. PC represents LTR retrotransposons specific probe (25 Pg) used as a positive control. Different lane numbers represent different transformant plants

For identifying siRNAs expression of *PHRE1* and *PHRE2,* Northern analysis in selected *Arabidopsis* T1 lines under heat stress resolved production of 22–24 bp siRNAs specific to *PHRE1* and *PHRE2*. The results were negative for the CaMV35s promoter-driven plants. The siRNAs isolated from wildtype control plants did not show any hybridization signal with their respective probes (Fig. 6). However, higher expressions of *PHRE1* and *PHRE2* were detected by qRT-PCR analysis in leaves of transgenic lines compared to control plants (Fig. S4).

### *PHRE1* and *PHRE2* retroelements show interactions with TFs

Several TFs were predicted by scanning the JASPAR database such as TCP20, DOF2, DOF ZFP, DOF PBF, MYB1, WRKY40, MYB24, KANADI, WRKY18, MYB81, MYB119, GATA, DOF53, DOF57, MYB1, TCP4, TCP8, ethylene response 1, MYB113, NAC083, and MYB33, and their homologs were characterized from the moso bamboo genome database (Table S2)*.* The qRT-PCR for these TFs, resulted in only three showing significant differential expression in heat stressed plants than control plants. Assayed in root, leaf, and stem tissues collected under normal and heat stressed plants, statistically consistent and significant down-regulation of the target transcripts of three TFs, TCP20, DOF2, and GATA could be independently achieved in leaves and roots of plants subjected to heat-stress (Fig. S6). Our results suggested that TCP20, DOF2, and GATA are involved in the regulation of heat stress tolerance and host adaptation to environmental stress.

**Fig. 7:**
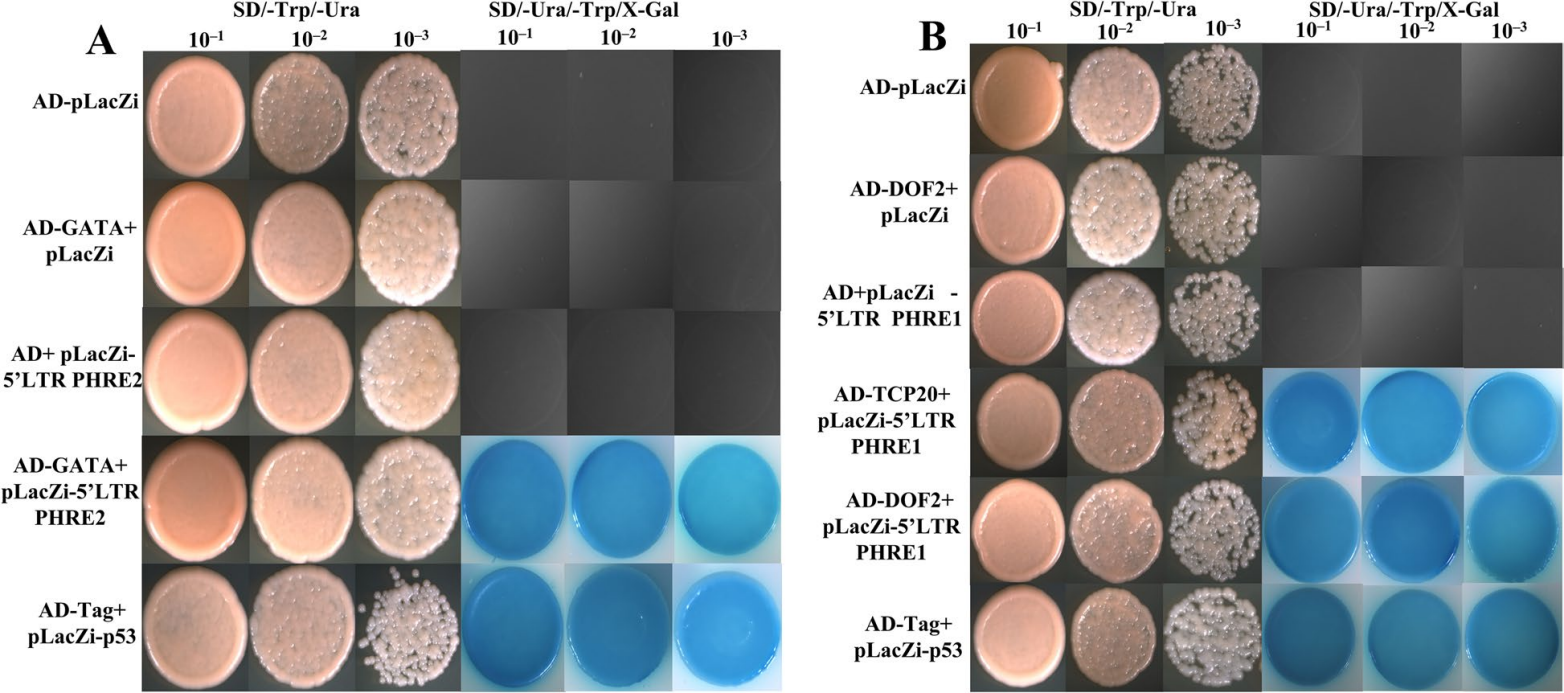
The yeast one-hybrid assay showing TCP20, DOF2, and GATA transcription factors (TFs) interactions with *PHRE1* and *PHRE2* LTR. The bait constructs pLacZi -*PHRE1,* pLacZi -*PHRE2,* and prey constructs pGADT7- TCP20/DOF2, pGADT7- GATA respectively were co-transformed into yeast strain EGY48, and the interactions were examined on SD/-Ura/-Trp/X-Gal plates. The blue color on the plate indicates the TCP20, DOF2, and GATA TF's interaction with LTRs. Plasmid pairs pGADT7-Tag + pLacZi - P53 acted as positive and negative controls. Each yeast colony was dissolved in 100μl of sterilized water and diluted 10^–1^ to 10^–3^. At least three colonies per combination were tested and analyzed

Since PHRE elements are also involved in heat stress response, our interest was to know whether any interactions existed between PHRE elements and TFs. Interestingly, in the yeast bait-prey assay, these TFs showed a specific pattern of interaction with PHRE elements. TCP20 (PH01001418G0330) and DOF2 (PH02Gene21543) were found to interact with *PHRE1* in yeast cells, which were able to grow on the minimal medium containing SD/-Ura/-Trp/X-Gal (Fig. 7A). They did not seem to interact with the *PHRE2* element. Whereas the GATA (PH02Gene06016) was the only TF that was found to interact with *PHRE2*, as the corresponding yeast cells could grow well on the minimal medium (Fig. 7B). The yeast transformants carrying negative control plasmids (AD, BD, AD-TCP20/DOF2/GATA plus, BD, AD plus BD-*PHRE1*/*PHRE2*) were not able to grow (Fig.7). These results confirmed the selective interaction of TCP20 and DOF2 with *PHRE1*, and GATA with *PHRE2*, further providing evidence for the promoter activity of 5’LTRs in *PHRE1* and *PHRE2* activation.

Based on the BiFC assay, strong GPF signals could be detected in the epidermal cells of pSPYNE-*PHRE1*: pSPYCE-TCP20, pSPYNE-*PHRE1*: pSPYCE-DOF2*,* and pSPYNE-*PHRE2*: pSPYCE-GATA (Fig. S7 and S8). No interactions were observed between the epidermal cells of tobacco leaves co-infiltered with negative controls.

## Discussion

The abundance of retroelements in the higher-order genome remains a biological mystery. Although they throw light on genome evolution, the elementary advantage of accumulating these elements remains unsolved. However, increasing evidence shows that these are involved in several homeostatic mechanisms including imparting stress tolerance [16, 26, 28, 46, 48]. Besides, they act epigenetically triggering transient gene expression associated with several biosynthetic pathways and regulatory elements. The biological role of the most common retroelement, the LTR retrotransposons, seems to be intricate and is majorly derived from its property of reverse transcription of their genomic RNA. Experimentally induced variants of LTR retrotransposon insertion confer stress responsiveness to nearby genes. Mobility bursts may occur which can generate novel, or alter stress-responsive regulatory gene networks [51, 52]. The stress-induced activation of LTR transposon has been shown to increase transgenerational transposition [53, 54]. The transcriptional activation of LTR retrotransposon is regulated by a siRNA-related pathway, post-transcriptional modifications. These retroelements comprise trans-acting siRNAs, which are responsible for gene regulation at the transcriptional and post-transcriptional levels [20, 24, 55, 56]. LTR retrotransposons potentiate a unique balance by orchestrating tight stress regulation of physiological processes of plant growth and development. In deciphering the role of transposable elements, we make constant efforts to engineer/improve stress resistance in economically important crops. In this pursuit, we studied moso bamboo to decipher the role of two native LTR retrotransposons, *PHRE1* and *PHRE2*, and their involvement in environmental stress, particularly heat stress.

We have used different biological systems to study the effect of *PHRE1* and *PHRE2* by estimating tissue-specific expression levels and their potential interactions with other genetic factors. As mentioned previously, moso bamboo is monocarpic and shows very infrequent sexual reproduction [57], due to its long vegetative growth and delayed flowering intervals. This pushes moso bamboo far behind in employing key bio-protocols that have been perfected in model systems such as *Arabidopsis*, rice, and tobacco. For instance, the development of transgenic plants by various genetic transformation techniques is technically difficult and extremely impractical in moso bamboo including micropropagation, in planta transformation by agroinfiltration, vacuum infiltration, floral dip, sonication, and gene delivery spraying [48]. Given this inconvenience, we have used SWNT transformation to deliver the LTR retrotransposons plasmid DNA into moso bamboo plants without transgene integration [50]. We could find that the internalization of nanoparticles in the transformant cells produced enhanced *GFP* expression levels in the leaves after 72h. Recently several reports have demonstrated carbon nanoparticles as efficient delivery systems of plant biomolecules such as DNA, RNA, and protein and are capable of strong internalization in planta [49]. The carbon nanotubes enable plasmid delivery without transgene integration into crop species [58–60], which are expressed across different tissues including leaves, roots protoplast, and immature tissues [59]. Enhanced *GFP* expression in leaf protoplasts through carbon nanotube delivery has been reported in wheat (*Triticum aestivum*) and arugula (*Eruca sativa*) [50]. Also, the use of nanoparticles mediated transformation has been demonstrated for siRNA production to silence genes [61, 62].

To demonstrate the *in vivo* expression pattern of *PHRE1* and *PHRE2* elements we have primarily used *Arabidopsis*, for transformation. The study revealed that the 5’LTRs of both the elements could show promoter activity in driving the expression of the *GPF* gene in *Arabidopsis*. The promoter activity of LTRs has been reported by several studies in different plant species [52, 63]. These results are in agreement with previous reports of greater promoter activity of the LTR in transgenic *Arabidopsis* [64, 65]. Takeda *et al.* (1999) demonstrated that tobacco *Ttol* promoter is responsible for enhanced expression patterns in transgenic tobacco lines by various stress. Remarkably, the specific role of 5’LTR as a promoter could be observed in transcriptional activity under heat stress. Also, both the 5’LTRs promoters activated the transcription and transposition of *PHRE1* and *PHRE2* in the transgenic plants. When we overexpressed both *PHRE1* and *PHRE2* in moso bamboo using SWNTs transformation, a similar GPF expression could be noticed in the leaf tissues. Additionally, *GUS* activity also was found activated by the 5’LTR promoters in both *Arabidopsis* and moso bamboo. The promoter activity of 5’LTR was higher than CaMV35s, and no transposition activity could be detected driven by CaMV35s promoter. These results were subsequently proved by the quantitative gene expression studies, which showed a parallel pattern. Besides, elevated expression of 5’LTR, *gag*, *pol*, and 3’LTR of *PHRE1* and *PHRE2* indicated that the entire retroelements were activated in the heat-stressed plants than in the control plants. This suggested that heat stress could induce the increased transcriptional activity of LTR elements, and thereby increased transposition. Recent reports indicate the efficiency of LTR retrotransposons is achieved by copy number and transposition, under hormonal regulation and irradiation stress [48]. The moso bamboo seedlings generated by tissue culture expressing *PHRE2* attained a significant increase in the copy number, suggesting its capability for retrotransposition [48]. Matsunaga *et al.* (2012) reported that *Arabidopsis* LTR retrotransposon *ONSEN* was inserted in the progeny of heat-stressed lines impaired in siRNAs demonstrating its role as heat stress promoter. Varying expression levels of LTR retrotransposons among different generations of transgenic lines, specifically the elevated expression 5’ and 3’ LTR in leaves, exemplified that *PHRE1* and *PHRE2* were stably integrated into the *Arabidopsis* genome.

We localized the expression of LTR retrotransposons in primary and lateral roots and matured leaves of moso bamboo. The enhanced expression in roots and leaves perhaps indicates the stress response role of these elements, because these organs are primary sensors of extraneous abiotic stresses, particularly heat, drought, salinity, etc. Tissue expression patterns indicated that *PHRE1* was abundantly expressed in the cortex root cells but spread throughout the roots and leaves. *PHRE2* was, however, predominantly expressed in roots associated with xylem and xylem parenchyma cells and in the guard cells of matured leaves. Our findings in moso bamboo draw parallels to the earlier reports of *Gypsy* retroelements in *Brachiaria* forage grasses [66]. Several LTR retrotransposons of the Ty3/*Gypsy* and Ty1/*Copia* family have been reported to predominantly be expressed in the sugarcane genome, suggesting that it has a specific crucial role in genetic variations, genome evolution, and adaptation to environmental stress [67].

We could detect abundant production of siRNA by these LTR retrotransposons, which was particularly high under heat stress. In biological systems, siRNAs are produced to regulate gene silencing and are involved predominantly in epigenetic processes [68]. The siRNA activity to regulate heat stress tolerance has already been reported in several systems such as *Arabidopsis* [26, 28, 69] mangrove [70], and Brassicaceae [27]. We also could observe the production of specific siRNAs in progeny lines. Heat stress significantly induced a higher accumulation of *PHRE1*-siRNA than *PHRE2*-siRNA. Transcriptional analysis indicated that moso bamboo LTR retrotransposons derived siRNAs (21–24 bp) might be involved in the transcriptional regulation of host genes [56]. In *Arabidopsis*, LTR retrotransposons in the chromatin gene *DDM1* are activated by mutations and produced 21–22-nt siRNAs involved in the regulation of epigenetic modification [71]. A recent study showed that LTR retrotransposons derived 24-nt siRNAs in *Rhizophora apiculate* could contribute to the progression of epigenetic TE silencing to maintain genetic diversity and thus an evolutionary response to stress conditions [70].

In addition, we confirmed LTR retrotransposon's interactions with different TFs (TCP20, DOF2, and GATA) confirming their intrinsic roles in plant growth regulation. Various TF gene families such as TCP [72], MYB [73], WRKY [74, 75], DOF (DNA-binding with one finger) [76] and GATA [77] have been analyzed in moso bamboo for their potential stress resistance functions. A recent analysis showed that heat shock TFs are involved in moso bamboo growth [78]. In our study, LTRs of *PHRE1* interacted with two different TFs such as *Ph*TCP20 and *Ph*DOF2, while LTRs of *PHRE2* interacted with *Ph*GATA, revealing its significant role in molecular function. The plant-specific TCP proteins contain a DNA binding domain (GGNCC), that plays a crucial role in the stress-associated regulation of plant growth and development [72, 79]. DOF proteins, comprising the DNA-binding C2C2-zinc domain, is the critical transcriptional regulator in plants for the different biological process including the regulation of plant growth and development in response to abiotic stress [76, 80]. Similarly, the GATA proteins with a CX2CX18CX2C Zinc finger DNA-binding domain are involved in the regulation of plant hormone signal transduction and response to environmental stress [77, 81]. The quantitative analysis of overexpression of TFs in response to heat stress in moso bamboo leaf, root, and stem, revealed significant downregulation of *Ph*TCP20 in the matured leaves, compared to the other two tissues. These results were consistent with a previous study that demonstrated the *Pe*TCP gene transcripts were significantly downregulated after the salicylic acid (SA) treatment in moso bamboo [72]. A similar downregulation, but in roots could be seen for *PhDOF2* and *PhGATA* after exposure to heat. Wang *et al.* (2016) reported significant differential expression patterns of four *DOF* genes associated with floral bud formation (*PhDOF4*, *PhDOF5*, *PhDOF20* and *PhDOF22*) under drought stress in moso bamboo plants, indicating positive regulation of the early stages of floral development. Downregulated expression patterns of *PeGATA26* in moso bamboo and *Arabidopsis* seedlings were observed under the gibberellic acid treatment [82]. We could establish these interactions in our study, which was further validated through tobacco BiFC assay, which showed that *PHRE1* and *PHRE2* were localized in the guard cells of tobacco epidermal leaf, and interaction TFs had occurred at the protein level. However, the precise mechanisms of interaction remain to be solved. Several TFs are known to have their binding sites associated with LTRs, regulating cell specific gene expression [46]. In retroviral systems such as HIV1, specific interaction of regulatory elements to LTR domain in activating transposition machinery is reported [83]. In other viral systems, such as in the rice tungro virus, TF interactions with promotor regions are also known to occur [84]. Moreover, direct interactions of TFs with *pol* [85] and *gag* [86] have also been reported in HIV1. It would require additional explorations to know what precise regulatory mechanisms *PHRE1* and *PHRE2* undergo in moso bamboo. Barring the mode of interaction, our results and reported interactions suggest that transposon activity occurs unfettered in moso bamboo under stress such as heat, which drives regulation of developmental processes, and conferring abiotic stress tolerance as well.

**Fig. 8:**
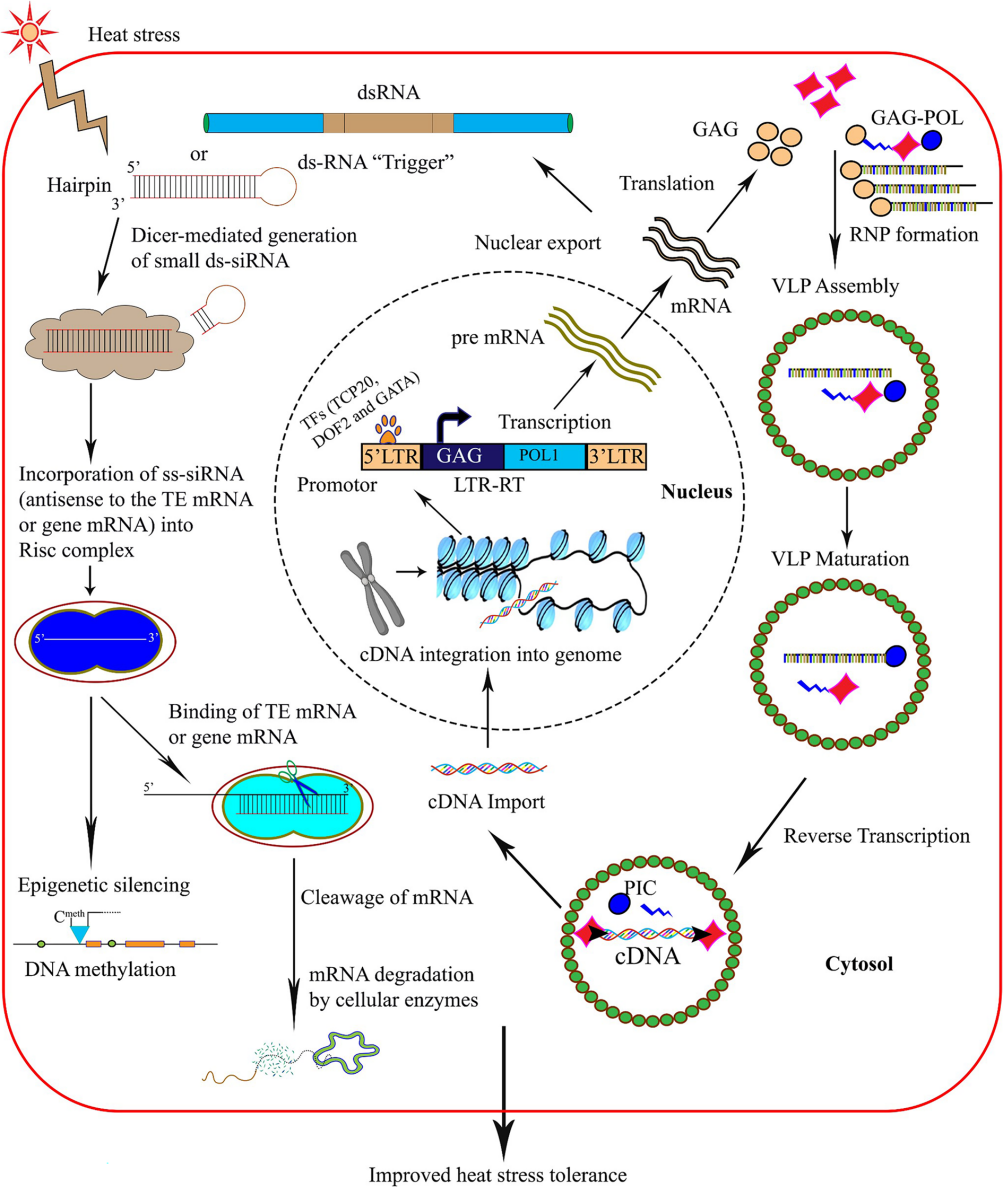
LTR retrotransposons regulate heat stress tolerance in moso bamboo. When transgenic moso bamboo seedling treated with heat stress, heat-dependent transcription factors, such as TCP20, DOF2, and GATA, bind to the LTR promoter of *PHRE1*/*PHRE2* to initiate transcription of mRNA from the *PHRE1*/*PHRE2*. In the cytoplasm, the mRNA encodes GAG and POL proteins which make virus-like particles (VLP) with reverse transcriptase to produce cDNA of *PHRE1*/*PHRE2*. Then, this cDNA is imported into the nucleus and integrated into the genome. LTR retrotransposons are also involved in epigenetic TE repression. The hairpin structure double-stranded RNA is produced from the mRNA of *PHRE1*/*PHRE2*. The dicer enzyme cleaves the double-stranded RNA into 21–25 nucleotide long siRNAs. Then, the siRNA was loaded onto the RISC complex where one strand is degraded and the other strand works as a template (antisense to the TE mRNA or gene mRNA) to post-transcriptionally silence the mRNA expression or this complex is transported to the nucleus, to initiate the RNA-directed DNA methylation (RdDM), to epigenetically induce transcriptional gene silencing or transposable elements

Based on the results, we propose an activity flow for the *PHRE* elements in moso bamboo under heat stress (Fig. 8). On exposure to heat stress, the signals for the stress are activated in the leaves and roots. On stress sensing, the stress-dependent TFs, *Ph*TCP, *Ph*DOF2, and *Ph*GATA bind to the 5’LTR of the PHRE elements and initiates the mRNA transcription. The entire retrotransposon sequence is transcribed and exported to the cytoplasm where the GAG and POL proteins produce virus like particles (VLPs). The reverse transcription follows to produce cDNAs of the complete retrotransposon which is imported back the nucleus to initiate transposition. By this activity, the copy number of the *PHRE* elements increases. Additionally, an epigenetic activity occurs when the mRNA forms a hairpin structure of double-stranded RNA, which is spliced by a dicer enzyme to 21–25 nucleotide long siRNAs. The siRNAs are subsequently loaded onto an RNA-induced silencing complex (RISC) where one of the strands gets degraded and the other acts as a template to silence mRNAs through pairing to the sense strand. Besides, RISC can also be imported to the nucleus to initiate RNA-directed DNA methylation (RdDM), which can induce specific silencing of the genes through epigenetic activity (Fig. 8). The specific silencing of genes could impart stress response in plants wherein certain metabolism is altered to offer increased protection to plant systems while conserving energy and resources.

## Conclusion

Plant LTR retrotransposons are directly linked to genome evolution and integrity as well as connected to different stress responses, but their natural behavior remains unclear. In this study, we have explored the occurrence, functions, and interactions of two moso bamboo LTR retroelements, *PHRE1* and *PHRE2*. Assaying using different biological systems, we could find that 5’LTR regions of both the elements show promoter activity and stress activation. A conspicuous transcription and transposition activity could be observed under heat stress. This could be related to their tissue specific expression patterns, as both are found in highly expressed roots and leaves, two major stress sensing organs in the plants. Under stress, the LTR element released several siRNAs indicating a role in epigenetic control. In addition, we could observe specific interactions with different TFs (*Ph*TCP20, *Ph*DOF2, and *Ph*GATA), which too was tissue specific. We could conclude that the *PHRE1* and *PHRE2* elements in moso bamboo, play several genetic roles such as promoter activity, transposition, tissue specific expression, epigenetic and gene-to-gene interactions while being activated significantly under stress. Also, the activity of these elements seemed largely dependent on adverse environmental factors. Taken together, the transcriptional activity of moso bamboo LTR retrotransposons provides a strong impetus for host adaptation to heat stress and their role as master regulators of the heat stress response.

## Material and Methods

### Moso bamboo and *Arabidopsis* plant materials and growth conditions

Moso bamboo seeds (*P. edulis*) were collected from the host institute, Zhejiang Agriculture and Forestry University (30°14′N, 119°42′E) in Lin’an, Zhejiang province, China. Seeds were germinated and plants were grown under greenhouse conditions. Fresh seeds picked from a single plant were surface sterilized with 70% ethanol for two minutes and washed with sterile distilled water. Subsequently, they were treated with 1% sodium hypochlorite (NaClO) for eight minutes and washed with sterile distilled water to remove the sterilant. Later, the seeds were germinated in pots containing soilrite. The seedlings were maintained at 24–25°C for 30–45 days under a 16:8h light: dark photoperiod. To analyze the heat stress response, five-week-old seedlings were incubated in a programmed growth chamber at 45°C for four hours with 70% RH, and a 16:8h light: dark photoperiod. The seedlings grown under 24–25°C conditions were set as the control. After four hours, the plant samples were collected, flashed by freezing in liquid nitrogen and were stored at –80°C for subsequent experiments.

Likewise, *Arabidopsis* seeds were surface sterilized in 70% ethanol for 10 min and washed with sterile water 5 times. The seeds were germinated in a petri dish containing half-strength Murashige and Skoog (MS) medium (Coolaber, China). The Petri dishes were incubated in dark at 4°C for 3 days and then transferred to a growth chamber. After 14 days, the healthy seedlings with well-established roots were transplanted into pots containing sterilized soil and soilrite mixture (2:1). The seedlings were watered on alternative days and maintained in a growth chamber at 24–25°C with 70% RH, and a 16:8h light: dark photoperiod. For heat stress treatment, a portion of the four-weeks old seedlings was exposed to 37°C for 24h in another growth chamber with 70% RH, and a 16:8h light: dark photoperiod. The remaining unexposed seedlings were used as controls. After 24h, the plant tissues were collected, flashed by freezing in liquid nitrogen and were stored at –80°C.

### Isolation of *PHRE1* and *PHRE2*

Full-length target sequences of *PHRE1* and *PHRE2* were identified in the moso bamboo genome using LTR-STRUC software with default parameters as depicted in our previous work [47, 48]. To clone *PHRE1* and *PHRE2*, genomic DNA (500 ng) was extracted from fresh leaves using a plant genomic DNA kit (Tiangen, China) following the manufacturer’s instructions. Later, *PHRE1* (4.98 kb; Fig. S9A) and *PHRE2* (5.51 kb; Fig. S9A), were amplified from the DNA using Phanta Max Super-Fidelity DNA Polymerase enzyme (Vazyme, China) with sequence-specific primers (Table S3) as per the manufacturer’s protocol. Subsequently, these fragments were cloned into the pUC18 vector, and the sequence insertion was ascertained by the Sanger sequencing method with different sets of primers (Table S4).

### *PHRE1* and *PHRE2* constructs for genetic transformation

Full-length 5’ LTR and open reading frame (ORF) sequences of *PHRE1* with β-glucuronidase (*GUS*) reporter gene, and 3’ LTR sequence of *PHRE1* (5’LTR+*gag*+*pol*+*GUS*+3’LTR; a total of 8.3 kb) were fused by overlap PCR. The final product was added between the attB1 (upstream) and attB2 (downstream) terminal ends by PCR. Primer details are given in Table S3. The attB-flanked *PHRE1* was sub-cloned into an entry vector pDONR207, using BP clonase enzyme (Invitrogen). These fragments were subsequently cloned into two binary vectors, pMDC164 harboring no promoter, and pMDC43 harboring CaMV35s promoter, using recombination-based Gateway cloning technique, mediated by the LR clonase enzyme (Invitrogen). A similar cloning strategy was used for *PHRE2* (5’LTR+*gag*+*pol*+*GUS*+3’LTR; a total of 9.3 kb) as mentioned above. The vector pMDC43 had a green fluorescent protein reporter gene (*GFP*) after the CaMV35s promoter, but the *GFP* was cloned between attB2 and hptII sites in the pMDC164 vector. Then, the recombinant clones were transformed into *E. coli* DH5α competent cells. Target sequence orientation was confirmed by PCR assay using *PHRE1* and *PHRE2* specific forward and reverse primers (Table S3). To ensure the orientation of these inserts, only positive clones were sequenced by the sanger’s method using different sets of primers.

### Transformation of *PHRE1* and *PHRE2* constructs into *Arabidopsis* plants

Four different recombinant gateway constructs such as pMDC164:*PHRE1*, pMDC43:*PHRE1*, and pMDC164:*PHRE2*, and pMDC43:*PHRE2* were individually transformed into *Agrobacterium tumefaciens* (LBA4404) competent cells by electroporation, and positive clones were selected using kanamycin according to a reported protocol [87, 88]. Genetic transformation in *Arabidopsis* was achieved by floral dip method, wherein *PHRE1* and *PHRE2* were transformed into fully blossoming plants using *Agrobacterium* containing pMDC164 and pMDC43 vectors [89]. Transgenic plants were maintained in a growth chamber at the previous conditions, and after 30 days, transgenic seeds were collected and sterilized as described. The pMDC164 and pMDC43 have hygromycin phosphotransferase (hptII) gene as a selectable marker, and a minimum inhibitory concentration of 30 μg/L of hygromycin was used to screen the T1 plants*.* Seedlings that developed new shoots and survived in the minimum inhibitory concentration, were maintained in a growth chamber for two weeks and in a half-strength Hoagland medium. Transgenic plants with well-established roots and shoots were transferred to pots containing sterilized soilrite mixture for further experiments. *Arabidopsis* plants transformed with *Agrobacterium* containing empty vector were used as control.

### Transformation of *PHRE1* and *PHRE2* constructs into moso bamboo

We have used an efficient carbon nanotube (CNT) diffusion method [50] to transform moso bamboo seedlings with *PHRE1* and *PHRE2* constructs. Before infiltration to the leaf, *PHRE1* and *PHRE2* plasmid were mixed with polyethyleneimine (PEI)-single-walled carbon nanotubes (SWNT). Briefly, 30 mg of dry carboxylate (COOH)-SWNTs (Sigma) was weighed, and bath sonicated for 10 min at room temperature, followed by continuous 30 min probe-tip sonication at 10% amplitude resulting in dark black solution in an ice bath. The absorbance of SWNTs was measured at 632nm with an extinction coefficient of 0.36 L mg^-1^ cm^-1^. 2-(N-morpholino) ethanesulfonic acid (MES) hydrate (Sigma-Aldrich) buffer solution at pH 5.5 was added to react with 2mg of COOH-SWNTS solution. Later, the carboxylic acid activators, ethyl carbodiimide (EDC, Sigma-Aldrich), and N-Hydroxysulfosuccinimide sodium salt (NHS, Sigma-Aldrich) was added dropwise to COOH-SWNTS suspension and incubated at room temperature in a bath sonication for 15 min. The suspension was transferred into prewashed Amicon 100k centrifugal filters (Merk) and centrifuged at 300g for 8 min to remove the free EDC/NHS and biproducts. Activated COOH-SWNTS reacted with a cationic polymer, polyethyleneimine (PEI), overnight on the orbital shaker at 180 rpm. The PEI-SWNTs suspension was transferred into 100K centrifugal filters by centrifugation at 1000×g for 20 min. After repeated centrifugation, the absorbance of PEI-SWNTs was measured as described above. Typically, the 50mg/L concentration of PEI-SWNTs suspension was adjusted for the mass ratio of 3:1 for PEI-SWNTs: plasmid DNA, by diluting with MES delivery buffer (25 mM MES, 15 mM MgCl_2_ at pH 6) per infiltration. Before infiltration, PEI-SWNTs buffer solution was incubated with targeted DNA (plasmid) at room temperature for 30 min to form the DNA-PEI-SWNTs complex. After the incubation, the DNA-PEI-SWNTs suspension was infiltered using a needleless syringe onto the abaxial surface of moso bamboo leaf. After 48h, a small portion of leaf tissue was cut and observed through the confocal microscope for *GFP* fluorescence to monitor the DNA-SWNTs efficiency.

### Locating the *GUS* reporter in the transgenic plants

For identification of *PHRE1* and *PHRE2* functions under heat stress, the positively charged CNTs (PEI-SWNTs) were incubated with negatively charged plasmid DNA vectors, pMDC164 (without CaMV35s promoter), and pMDC43 (with CaMV35s promoter) containing *PHRE1* and *PHRE2* and *GUS* as the reporter gene. Then, the plasmid vectors -PEI-SWNTs were infiltrated into moso bamboo leaves as described. To analyze the *GUS* expression in the T1 plants, a *GUS* histochemical assay was performed. Briefly, surface-sterilized seeds were germinated on a half-strength MS medium. A minimum inhibitory concentration of hygromycin (30 μg/L) was used to screen the T1 plants. Four-week-old, germinated seedlings were incubated in a programmed growth chamber at 37°C for 72 h with 70% relative humidity (RH) and 16:8h light: dark photoperiod. Subsequently, seedlings were incubated overnight in *GUS* solution (Coolaber, China) at 37°C, followed by four times washing with 70% ethanol and sterile water, respectively. The *GUS* expression blue spots in tissue were photographed under the microscope without damaging the tissue. Similarly, *GUS* histochemical assay was performed with five-week-old transgenic bamboo plants. Control was the seedlings transformed with *Agrobacterium* with empty vector, grown and processed as the samples, however, without hygromycin selection.

### Quantitative gene expression among the transgenics

Genomic DNA was extracted from the harvested leaves of transgenic moso bamboo and *Arabidopsis* T1 lines using the cetyltrimethylammonium bromide (CTAB) method [90]. Putative transformants were confirmed by PCR assay using specific primer sets (5’LTR, *GUS*, 3’LTR, and hptII antibiotic marker) (Table S3). Amplicons were electrophoresed in 0.8% (w/v) agarose gel. Positive transgenics were subjected to a quantitative real-time PCR (qRT-PCR) assay. For this, total RNA was extracted from the harvested root, leaf, and stem using an RNAiso Plus reagent (Takarabio) following the manufacturer’s instructions. The reverse transcription into cDNA was carried out using a cDNA synthesis kit (Prime Script RT reagent Kit, Takara). The PCR assay was performed in a CFX96 Touch system (Bio-Rad) using TB Green Premix Ex Taq II (Takarabio). The 10 μl reaction mix for each sample was contained 1.5 ng of cDNA, 750 nM of each forward and reverse primer with 5.5 μl of TB Green premix. The amplification condition was set as a hot start of 95°C for 3 min followed by 40 cycles of 95°C for 15 s and 60°C for 1 min. For determining the specificity of amplification, a melt curve analysis or dissociation program was run at 95°C for 15 s; 60°C for 15 s followed by a slow ramp from 60 to 95°C. Cycle threshold (Ct) values of each sample were imported from CFX manager software version 2.3 (Bio-Rad, China). The reference genes used were *actin* for *Arabidopsis* and nucleotide tract-binding protein (*NTB*) for moso bamboo. Expression of 5’LTR, *gag*, *pol* and 3’LTR of *PHRE1* and *PHRE2* was quantified in different transgenic plants and was calculated as average ΔCt values, i.e., the difference between the Ct means of transgene and reference genes. Three independent biological and three technical replicates were run. Primer details are enlisted in Table S3.

### Molecular characterization of transgenes

Southern blotting was performed to analyze the transposition pattern of *PHRE1* and *PHRE2* in the PCR-positive transgenic and wild-type moso bamboo plants. Approximately 10 μg of genomic DNA from each line was digested overnight at 37°C with 50U Pac1 or *Hind*III (New England Biolabs-High fidelity) for single cuts in the T-DNA. DNA digests were electrophoresed and resolved on 0.8% (w/v) agarose gel followed by transblotting to the positively charged nylon transfer membrane (Sigma-Aldrich, Amersham) by capillary action in 20× SSC buffer (3M NaCl, 0.3 M sodium citrate, pH 7). The amplified products of 5’LTR of *PHRE1* and *PHRE2* generated from the respective pUC18 clones were used as a probe. Gel purified PCR products were labeled using a digoxigenin (DIG) probe synthesis kit (Sigma-Aldrich). Blot hybridization was carried out overnight in DIG Easy Hyb buffer solution at 42°C followed by washing twice with 0.5× saline sodium citrate (SSC) buffer at 65°C. Probe corresponding to the coding region of *PHRE1* and *PHRE2* that hybridized with genomic DNA on the nylon membrane was detected by alkaline phosphatase-conjugated anti-DIG antibody followed by chemiluminescent substrate reaction (Sigma-Aldrich). Finally, the blots were exposed to x-ray film (Fujifilm) and chemo doc imaging system (Bio-Rad, China) for one hour at room temperature.

For the detection of siRNAs derived from *PHRE1* and *PHRE2* in transgenic plants, northern hybridization was carried out. The siRNAs were extracted from the fresh leaves of the transgenic plants using the Nucleospin miRNA isolation kit (Takarabio) following the manufacturer’s instructions. Small RNAs (10 μg) were denatured by heating at 68°C for 5 min and electrophoresed by resolving on 15% denaturing polyacrylamide gel followed by transblotting on a positively charged nylon transfer membrane (Sigma-Aldrich, Amersham). For probing, a 445 bp PCR product of the 5’ LTR of *PHRE1* and a 480 bp of the 5’ LTR of *PHRE2* were used. Probes were labeled by PCR using a DIG probe synthesis kit (Sigma-Aldrich) as described previously [87]. Blot’s hybridization was performed using DIG easy Hyb solution at 37°C, followed by washing two times in 0.5× SSC buffer at 42°C. Chemiluminescence images were captured by using a chemo doc touch system (Bio-Rad) according to the manufacturer’s instructions.

### *In situ* localization of *PHRE1* and *PHRE2* transcripts

*In situ* RNA hybridization was performed to localize transcripts of *PHRE1* and *PHRE2* in roots and leaf tissue of moso bamboo. The cDNA fragments of 5’ LTR amplified from their respective pUC18 clones were used to synthesize DIG-labelled sense and antisense RNA probes by *in vitro* transcription. A 25 μl reaction mix with DIG-labelled dUTP (Sigma-Aldrich) provided with gene-specific forward or reverse primers was used for transcription (Table S3). Bamboo tissue fixation, permeabilization, probe hybridization, and detection were performed according to previously described methods [91, 92]. For root cross-section, roots were fixed in 4% paraformaldehyde at 4°C overnight, subsequently washed briefly with 0.1% phosphate buffered saline, and embedded in 5% ultra-low gelling regular agarose (Takara, Clontech). The sections were cut into 100 μm thickness using a Leica UC7 ultramicrotome (Leica Microsystems). Tissue specimens were mounted on glass microscope slides and examined in a Zeiss Imager M2m compound microscope.

### Analysis of transcription factors (TFs)

Using LTR sequences of *PHRE1* and *PHRE2*, TFs were predicted by querying the JASPAR 2020 database (http://jaspar.genereg.net/). The homologous genes of TFs were characterized from the moso bamboo genome database (http://www.bamboogdb.org). Total RNA was isolated from the leaf, root, and stem of bamboo transgenic plants treated with heat stress as well as from the normally grown. The RNA was reverse transcribed into cDNA and the qRT-PCR was carried out. Fold change of the TFs expression was calculated by augmented comparative Ct method using log_2_ transformed mean data [93]. *NTB* of bamboo was used as the reference gene. Primer details are enlisted in Table S3.

### Cloning and confirmation of LTRs and TFs in bait and prey vectors

Total RNA isolated from the fresh moso bamboo leaves using TRIzol Plus (Takara bio) was tested for quantity and quality using a Nanodrop ND-1000 spectrophotometer (Thermo Scientific). Approximately 400 ng of purified RNA was reverse transcribed using a cDNA synthesis kit (Prime script RT, Takara). Fragments of TFs, TCP20, DOF2 (DNA binding with one finger), and GATA were amplified separately from the bamboo cDNA and cloned into a pESI-T vector (Yeasen, China) using specific primers. Primer details are given in Table S3. Five microliters of the ligated product were transformed into 100μl of DH5α competent cells and selected on Luria broth (LB) plates with Ampicillin (50μg/ml). Positive colonies were confirmed by PCR, using M13 primers. To confirm the successful cloning of the TFs inserts, colonies were sequenced using M13 primers by Sanger’s method.

The 5’ LTR fragments of *PHRE1*, *PHRE2,* and TFs (TCP20, DOF2, and GATA) were initially amplified from the pESI-T clone with 15 bp overhangs at the upstream and downstream of the gene sequence, respectively, through PCR. Primer details are given in Table S3. To generate the bait construct, 5’LTR of *PHRE1* and *PHRE2* were individually cloned into *Kpn1* and *Xho1* sites of pLacZi, and the TFs (TCP20, DOF2, and GATA) were inserted downstream of Gal4 into pGADT7 as the prey construct (Fig. S9 B). Constructs were generated using the Clone press II One Step Cloning Kit (Vazyme, China). Five microliters of the ligated product were transformed into 100μl of DH5α competent cells and selected on LB plates with Kanamycin (50μg/ml). Positive colonies were confirmed by PCR using gene-specific primers (Table S3), followed by sequencing using CDS-specific primers. The sequence confirmed clone was transformed into yeast (*Saccharomyces cerevisiae*) competent strain EGY48 separately using high-efficiency polyethylene glycol (PEG)/LiAc-based method (Yeast transformation System2, Clontech, USA). Transformed yeast cells were selected on the minimal synthetic defined (SD) medium deficient in Trp (SD/-Trp) and Ura (SD/-Ura).

### Yeast one hybridization assay

Yeast one-hybrid assay (Y1H) was performed by co-transformation of yeast strain EGY48 competent cells with plasmids. Activation domain (AD), pLacZi, AD-TCP20+pLacZi, AD-DOF2+pLacZi, AD+5’LTR *PHRE1*, AD+5’LTR *PHRE2* were selected as negative interaction, while AD-TCP20+pLacZi -5’LTR *PHRE1*, AD-DOF2+ pLacZi -5’LTR *PHRE1*, AD-GATA+pLacZi -5’LTR *PHRE2*, and AD-Tag/ pLacZi - P53 were selected as a positive interaction. The combinations were grown separately at 30°C in 50 ml SD/-Trp and SD/-Leu, shaken at 180 rpm until OD600 reached 0.8 (16–20 h). Then cells were harvested by centrifugation at 1000×g for 5 min, and cell density was adjusted to >1×108 cells per ml. One milliliter of each AD and pLacZi cell culture was added to 48 ml of 2× yeast peptone dextrose adenine (YPDA) containing 50 μg/ml kanamycin and cultivated at 30°C at 50 rpm, examined under the microscope when zygotes appeared after 20–24 h. Cells were harvested by centrifugation at 1000×g for 5 min, the cell pellet was washed with 0.5× YPDA containing 50 μg/ml kanamycin and finally resuspended in 10 ml of 0.5× YPDA containing 50 μg/ml kanamycin. To calculate the mating efficiency, 100 μl of the mated culture (1/10, 1/100, 1/1000, and 1/10,000 dilutions) was spread on SD/-Trp, SD/-Ura, and SD/-Trp/-Ura agar plates and incubated at 30°C for 3–5 days. The remaining culture was plated on SD/-Trp/-Ura/X-Gal (40 μg/ml X-Gal) agar plates. The blue colonies that appeared on SD/-Trp/-Ura/X-Gal agar plates were streaked on selection plates SD/-Trp/-Ura/X-Gal and incubated at 30°C for 3–5 days.

### Bimolecular fluorescence complementation assay

*In-planta* interactions of TFs (TCP20, DOF2, and GATA) with the 5’LTR promoters of *PHRE1* and *PHRE2* by using bimolecular complementation fluorescence (BiFC) assay in *Nicotiana benthamiana* (tobacco) plants and assessed the interactions using confocal microscopy. The fragments of *PHRE1*, *PHRE2 (*5’LTR+*gag*+*pol*+3’LTR) were separately inserted into the pSPYNE (Fig. S9 C) and the TFs into pSPYCE plasmids. Ligation and transformation were performed using the ClonExpress II One Step Cloning kit as explained above. All the constructs were confirmed by sequencing before transformation into *A. tumefaciens* strain GV3101. Different pairs of constructs were made such as pSPYNE-*PHRE1*: pSPYCE-TCP20, pSPYNE-*PHRE1*: pSPYCE-DOF2, and pSPYNE-*PHRE2*:pSPYCE-GATA as positive sets, and pSPYNE:pSPYCE, pSPYNE-*PHRE1*:pSPYCE, pSPYNE:pSPYCE-TCP20, pSPYNE:pSPYCE-DOF2, pSPYNE-*PHRE2*:pSPYCE, and pSPYNE:pSPYCE-GATA were used as negative controls. To test protein interactions, each pair of constructs was co-transformed into the abaxial side of four-week-old tobacco leaves. The *GFP* was examined in the nuclei of epidermal cells of transformed tobacco leaves carrying different constructs. Images of fluorescence and 4,6-diamidino-2-phenylindole (DAPI) staining of transfected plants were taken using a Zeiss LSM 510 Meta confocal laser scanning microscope after 48 h dark treatment, as previously described [94]. The plants transformed with *A. tumefaciens* harboring empty vector were treated as control. The primers used for the BiFC assay are listed in Table S3.

## Supplementary Information

**Additional file 1.**

**Additional file 2.**

**Additional file 3.**

**Additional file 4.**

**Additional file 5.**

## Data Availability

Supplementary Data for this article are available
