# Supplementary material for: Long terminal repeats (LTR) and transcription factors regulate PHRE1 and PHRE2 activity in Moso bamboo under heat stress
Source: BMC Plant Biol. 2021 Dec 9;21:585. doi: 10.1186/s12870-021-03339-1 (PMC8656106; doi:10.1186/s12870-021-03339-1)
Supplement: Supplementary file 1 — Additional file 1. [file 12870_2021_3339_MOESM1_ESM.docx]

**Supplementary Information for**

**Long terminal repeats (LTR) and transcription factors regulate *PHRE1* and *PHRE2* activity in Moso bamboo under heat stress**

Pradeep K Papolu^1^, Muthusamy Ramakrishnan^1^, Qiang Wei^2^, Kunnummal K. Vinod^3^, Long-Hai Zou^1^, Kim Yrjala^1^, Ruslan Kalendar^4^, Mingbing Zhou^1,5 *^

^1^State Key Laboratory of Subtropical Silviculture, Zhejiang A&F University, Lin’an, Hangzhou 311300, Zhejiang, China

^2^Co-Innovation Center for Sustainable Forestry in Southern China, Nanjing Forestry University, Nanjing 210037, Jiangsu, China

^3^Division of Genetics, ICAR - Indian Agricultural Research Institute, New Delhi, India

^4^Helsinki Institute of Life Science HiLIFE, Biocenter 3, Viikinkaari 1, FI-00014 University of Helsinki, Finland

^5^Zhejiang Provincial Collaborative Innovation Centre for Bamboo Resources and High-efficiency Utilization, Zhejiang A&F University, Hangzhou, China

***Correspondence**

Professor Mingbing Zhou,

State Key Laboratory of Subtropical Silviculture,

Zhejiang A&F University, Lin'an, Hangzhou 311300,

Zhejiang Province, P.R. China.

Email: [zhoumingbing@zafu.edu.cn](mailto:zhoumingbing@zafu.edu.cn)


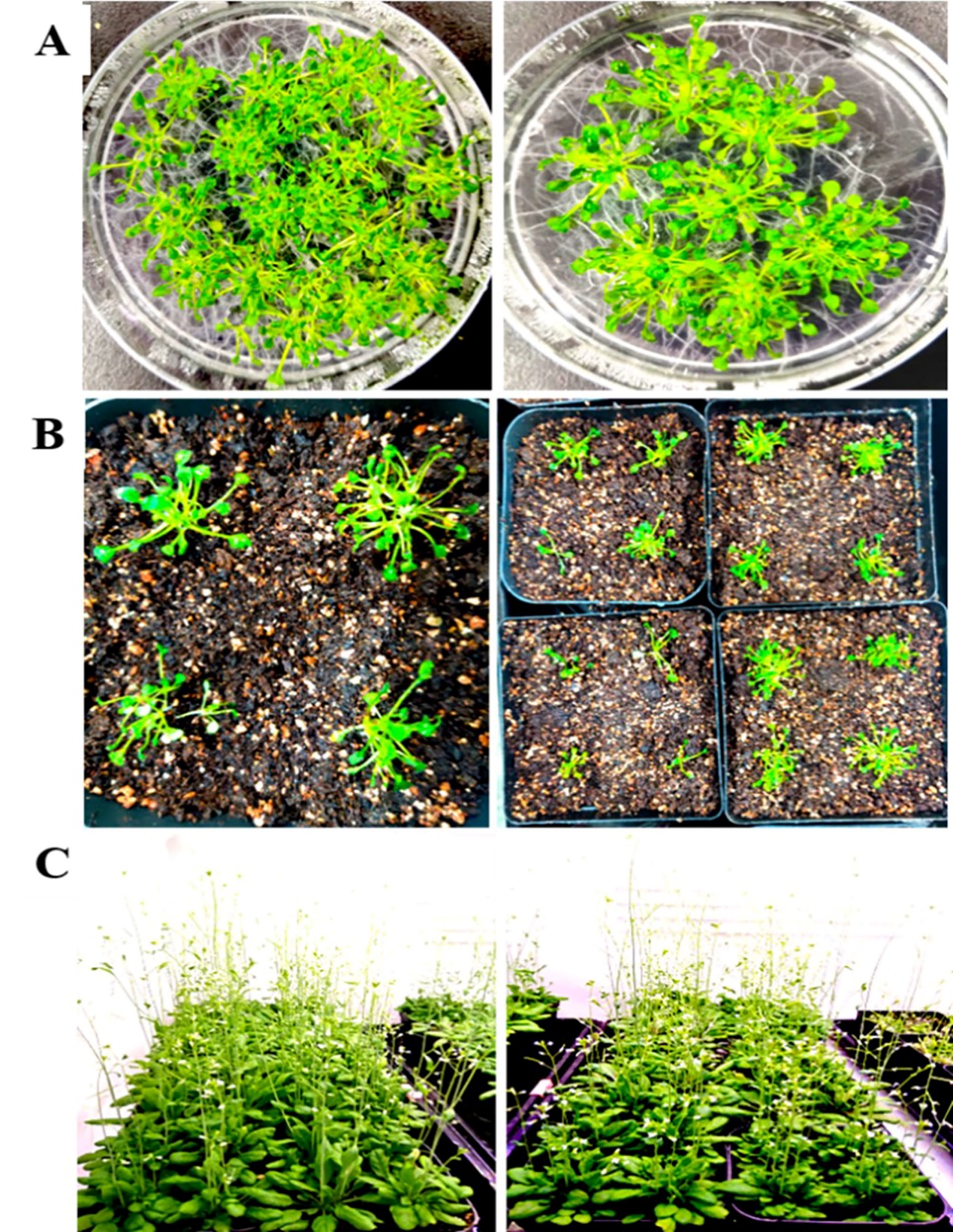


**Fig. S1. Generation of transgenic plants.** *Arabidopsis* transformed with moso bamboo *PHRE1* and *PHRE2* constructs by *Agrobacterium*-mediated floral-dip transformation. (A) Germination of sterilized seeds in MS medium and antibiotic marker screening of plantlets in soil supplemented with 30mg hygromycin, (B) Transformed plantlets that survived hygromycin selection, (C) Shoot induction and flowering of transformed plants in the growth chamber


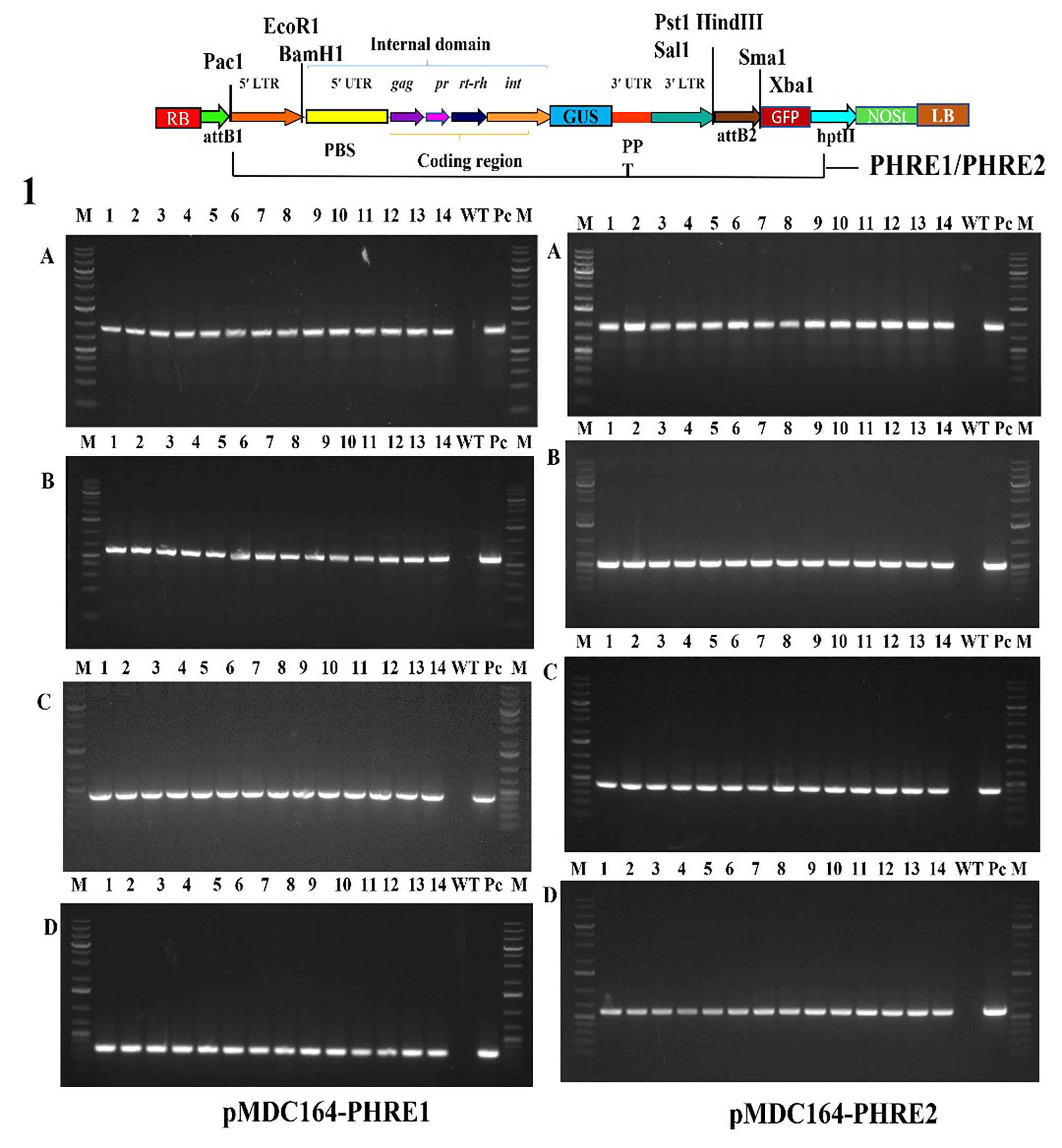


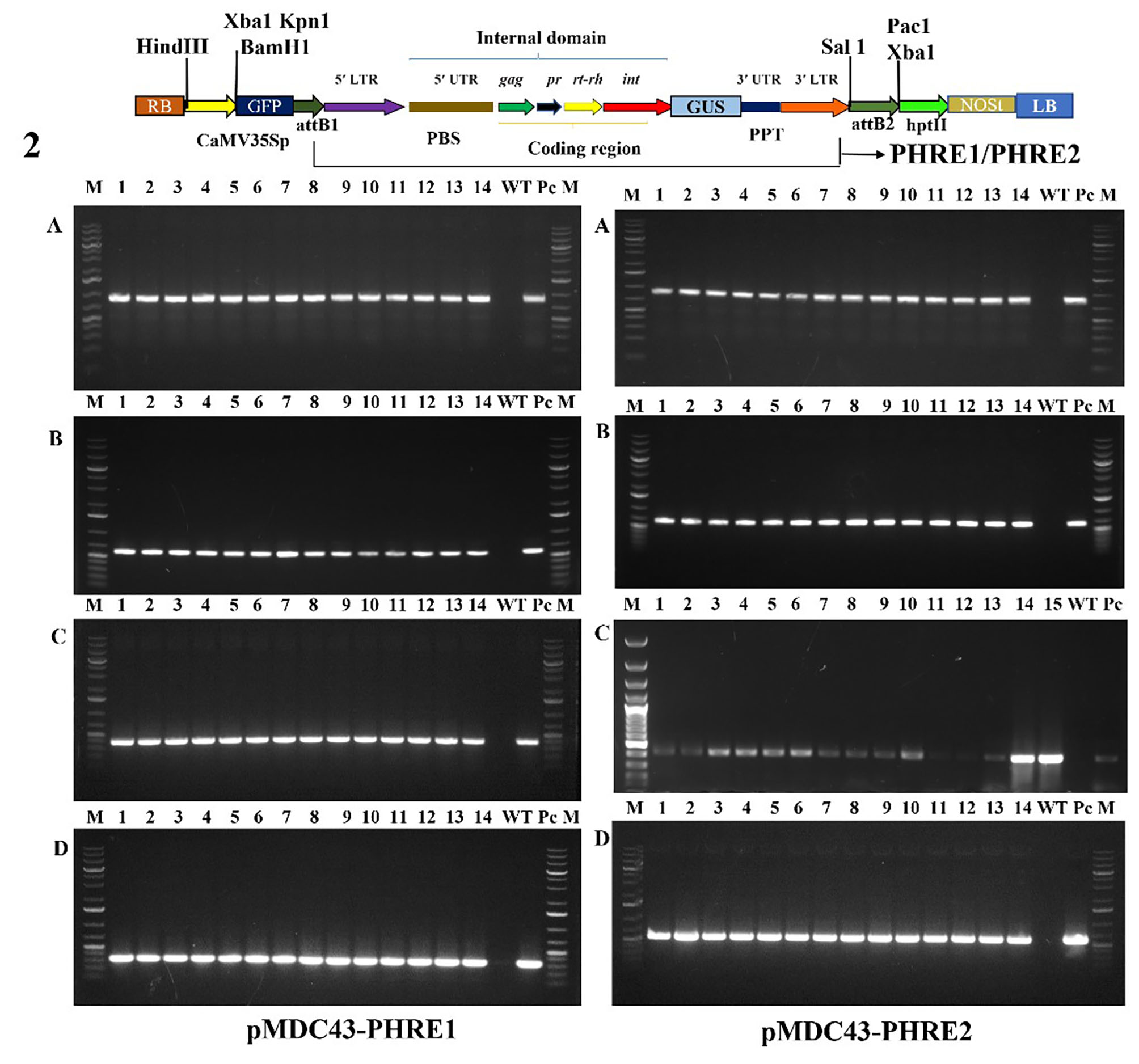


**Fig. S2. Schematic representation and orientation of *PHRE1* and *PHRE2* constructs**. Number 1 represents the pMDC164 vector. Number 2 represents the pMDC43 vector. The T-DNA region constitutes the full-length *PHRE1* and *PHRE2* flanked by attB1 and attB2 in a gateway cassette without 35s promoter. The GUS reporter gene, GFP, and Hygromycin resistance gene (hptII) were used as the selectable markers. Bottom panel images demonstrate the PCR confirmation of pMDC164-*PHRE1*/*PHRE2* and pMDC43-*PHRE1*/*PHRE2* in the progeny plants of different T1 *Arabidopsis* transgenic lines. Lanes-M: 1kb DNA marker, (A) Amplification of the 5'LTR using specific primers of *PHRE1*-left (658 bp) and *PHRE2*-Right (685 bp), (B) Amplification of GUS gene of *PHRE1*-left and *PHRE2*-Right (550 bp), (C) Amplification of hptII gene of *PHRE1*-left and *PHRE2*-Right (415 bp), (D) Amplification of 3’LTR *PHRE1*-left (380 bp) and *PHRE2*-Right (550 bp). WT – untransformed control or wildtype *Arabidopsis* plant and Pc – PCR products of the cloned gene as a positive control.


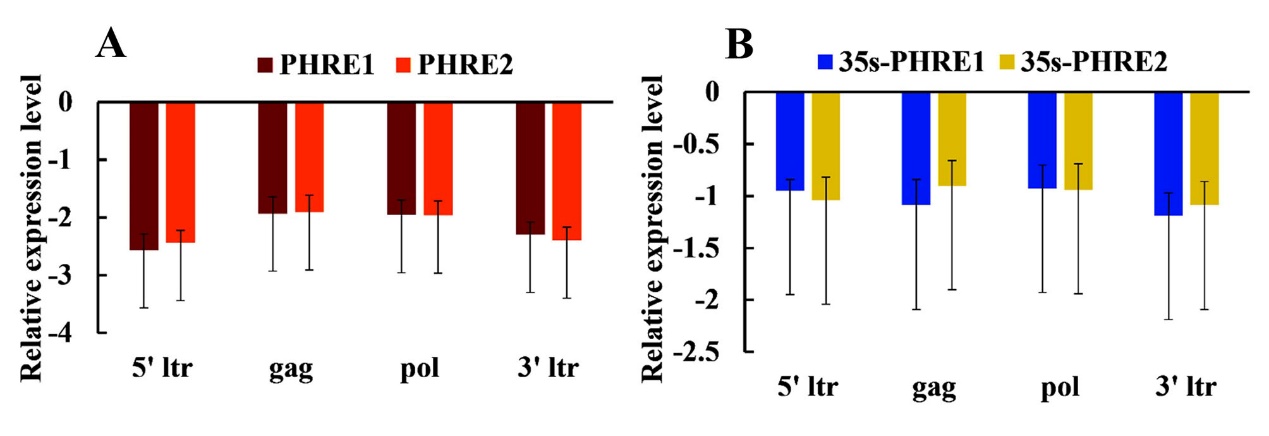


**Fig. S3. Detection of *PHRE1* and *PHRE2* expression in Moso bamboo transformants by RT-qPCR.** (A) Relative transcript levels of pMDC164-*PHRE1*/*PHRE2*, and (B) 35s-pMDC43-*PHRE1*/*PHRE2* (5’LTR, gag, pol, 3’LTR) in leaves quantified by the 2^-ΔΔCT^ method. In pMDC164, 5’ LTR acts as a promoter, and in pMDC43, 35s acts as a promoter. Retrotransposon expression was normalized with the Moso bamboo NTB gene. Each bar represents the log2 transformed mean of RT-qPCR runs in three biological and three technical replicates with standard errors.


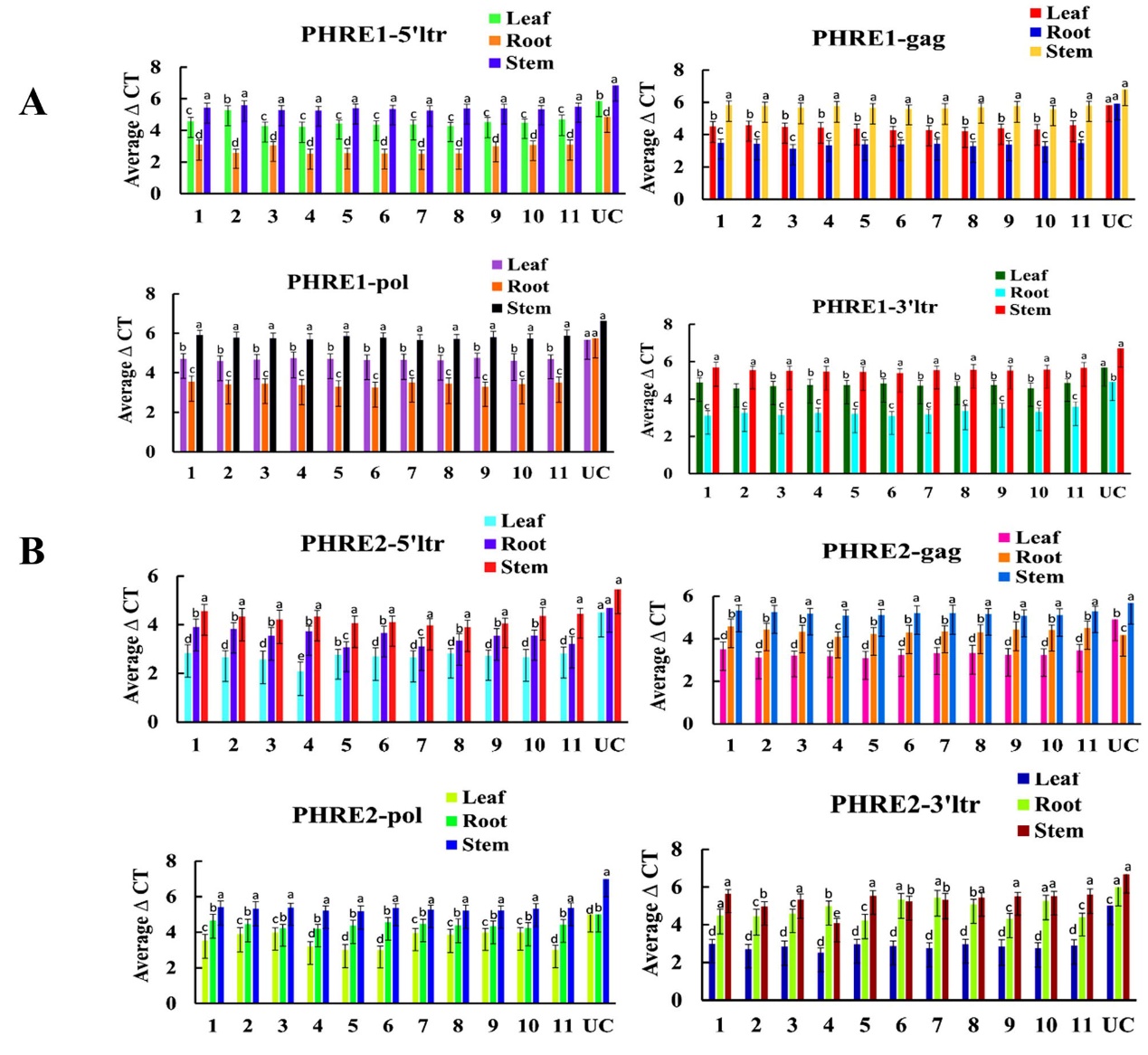


**Fig. S4. Detection of pMDC164:*PHRE1* and pMDC164:*PHRE2* in T1 *Arabidopsis* lines by RT-qPCR.** Relative transcript levels of *PHRE1*(A) and *PHRE2* (B) were expressed as ΔCt, which indicates the difference in CT mean of transgene and reference gene (*Actin* gene of *Arabidopsis*). Higher ΔCt values represent the lower expression of retrotransposon in the corresponding lines. Each bar indicates the mean with standard error derived from three independent biological and three technical replicates. Bars with different letters are statically different at P < 0.01, Tukey's HSD test. UC-un treated transgenic control plants.


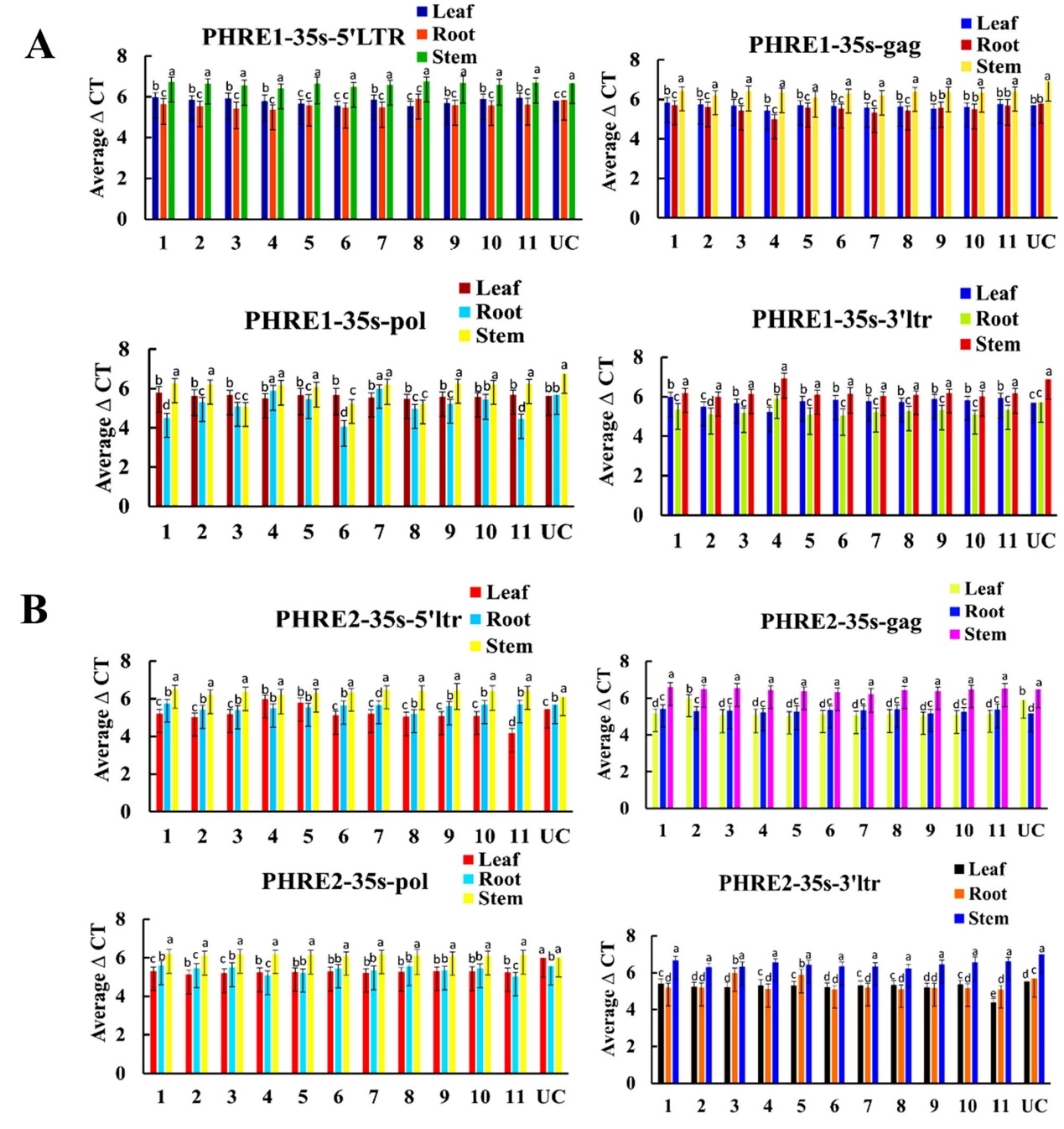


**Fig. S5. Detection of 35s-pMDC43:*PHRE1* and 35s-pMDC43:*PHRE2* in T1 *Arabidopsis* lines by RT-qPCR.** Relative transcript levels of (A) *PHRE1* and (B) *PHRE2* were expressed as ΔCt, which indicates the difference in CT mean of transgene and reference gene (*Actin* gene of *Arabidopsis*). Higher ΔCt values represent the lower expression of a retroelement in the corresponding lines. Each bar indicates the mean with standard error derived from three independent biological and three technical replicates. Bars with different letters are statically different at P < 0.01, Tukey's HSD test. UC-un treated transgenic control plants


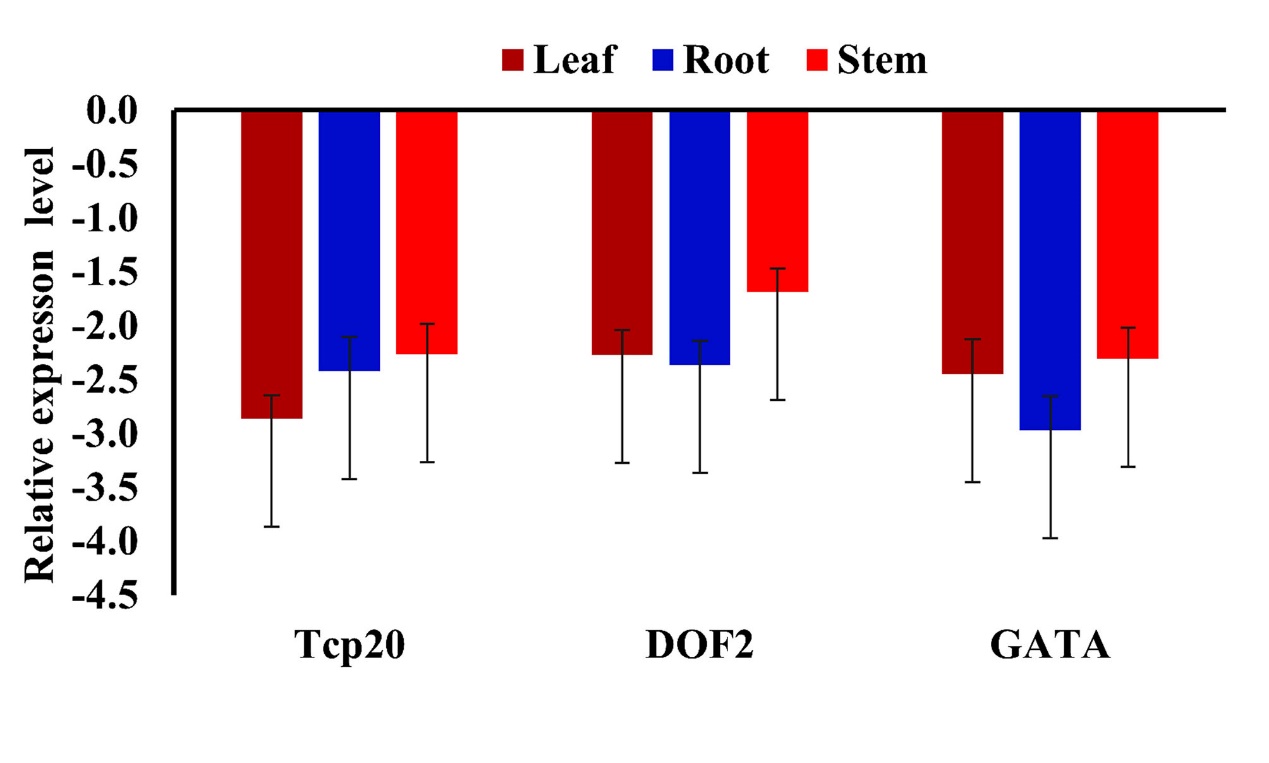


**Fig. S6. Relative transcript abundance of TCP20, DOF2, and GATA transcription factors in different tissues of Moso bamboo.** Using the transcript level in NTB as a reference, candidate genes (expression level was quantified by the 2^-ΔΔCT^ method) were significantly downregulated in root, leaves, and stem. Each bar represents the log2-transformed mean of quantitative reverse-transcription PCR (qRT-PCR) runs in triplicate with standard errors.


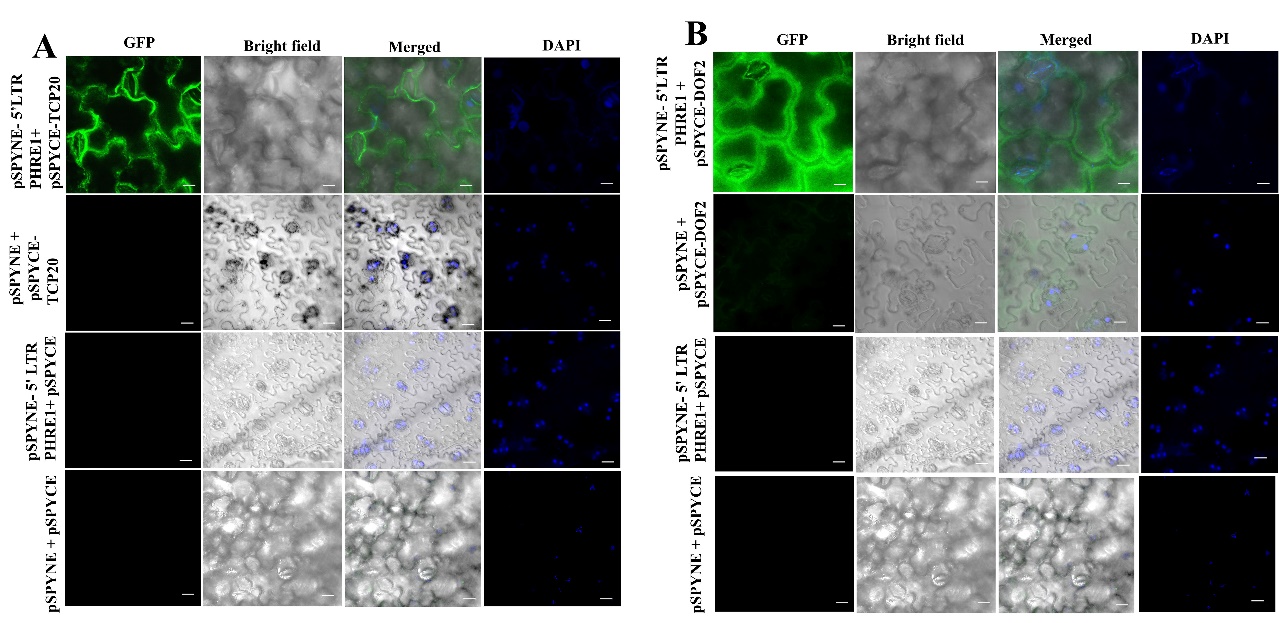


**Fig. S7. Bimolecular fluorescence complementation (BiFC) assay of *PHRE1* interaction with TCP20 and DOF2 observed in the periphery of the epidermal cell of tobacco leaves co-infiltrated with *Agrobacterium* harboring, A. pSPYNE-*PHRE1*; pSPYCE-TCP20 and B.** **pSPYNE-*PHRE1*; pSPYCE-DOF2 constructs**. Nuclei of tobacco guard cells were stained with 4, 6-diamidino-2-phenylindole (DAPI) and visualized under the confocal microscope. Scale bar represents 50 μm.


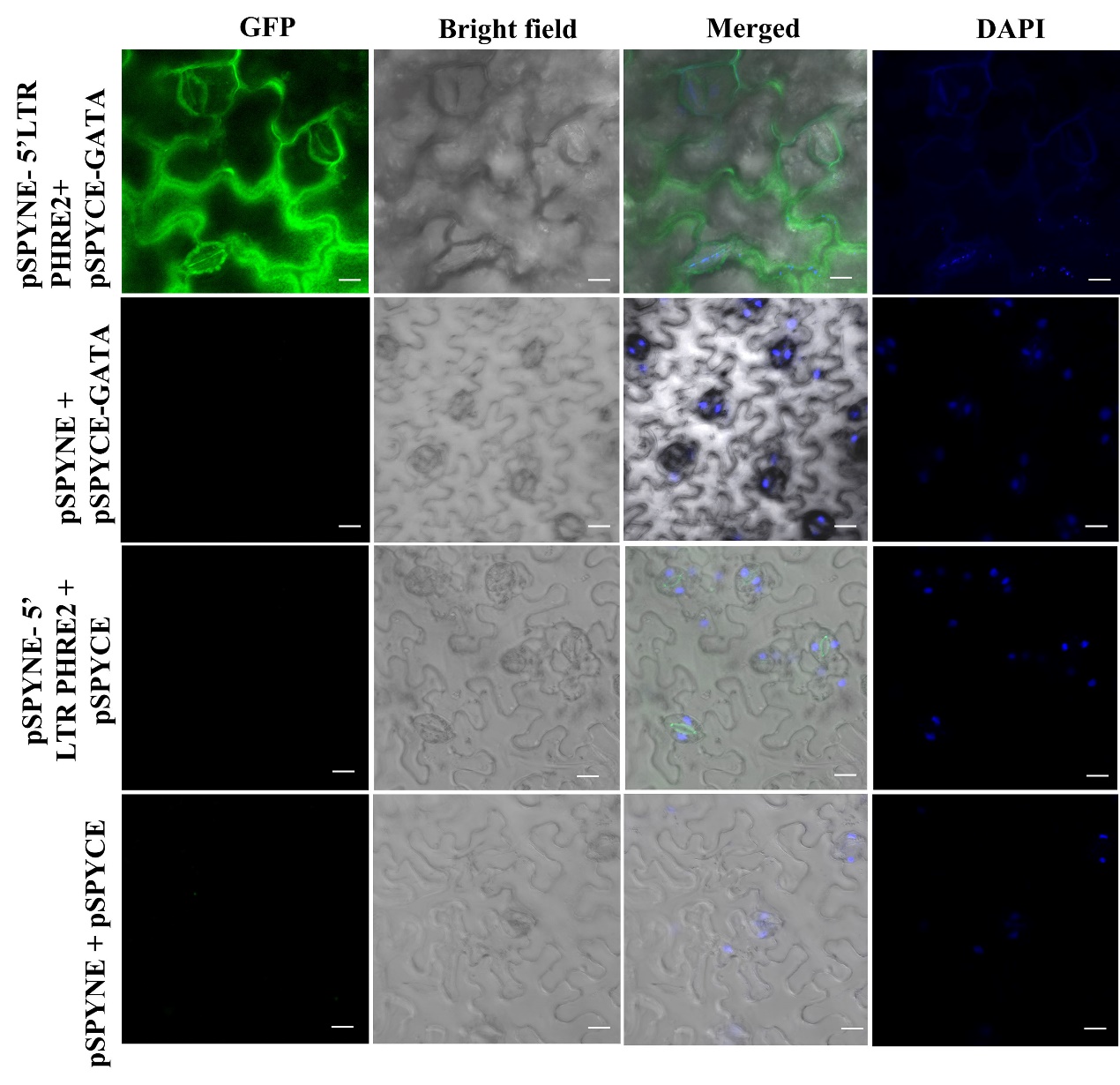


**Fig. S8. Bimolecular fluorescence complementation (BiFC) assay of *PHRE2* interaction with GATA observed in the periphery of the epidermal cell of tobacco leaves co-infiltrated with *Agrobacterium* harboring pSPYNE-*PHRE2*; pSPYCE-GATA constructs**. Nuclei of tobacco guard cells were stained with 4, 6-diamidino-2-phenylindole (DAPI) and visualized under the confocal microscope. Scale bar represents 50 μm.


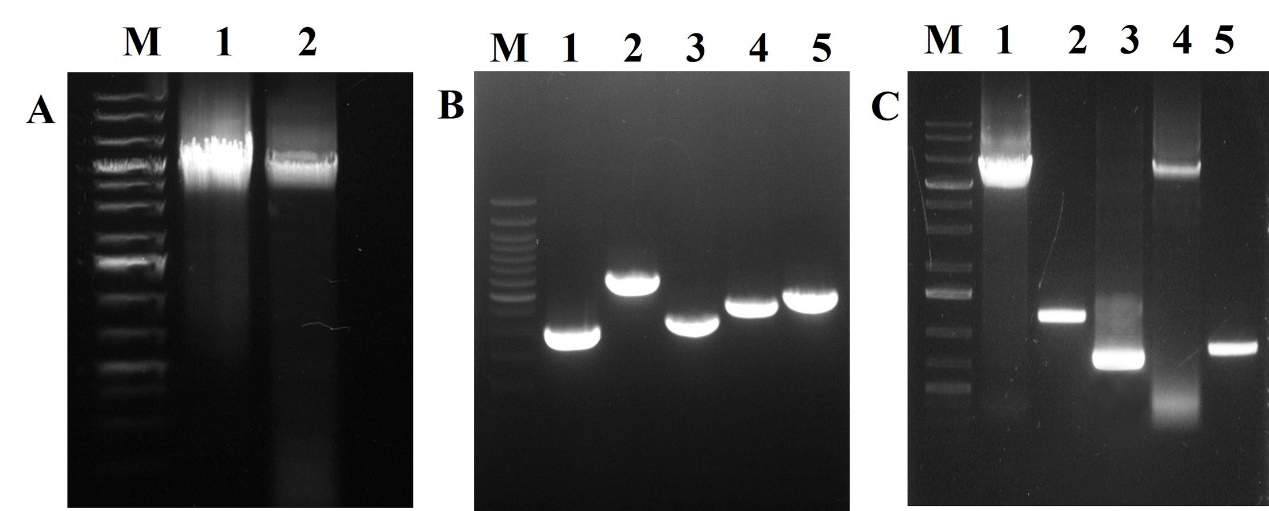


**Fig. S9. Amplification of Moso bamboo LTRs, and TFs for cloning in TA vector, Yeast vector, and BIFC vector.** Amplification of moso bamboo LTRs, and TFs for cloning in TA vector, Yeast vector, and BIFC vector. Lanes - M: 1kb and 100 bp DNA marker, **(A)** PCR amplified products for cloning in pUC18 vector. 1. *PHRE2* (5515 bp), 2. *PHRE1*(4980 bp). **(B)** PCR amplified products for cloning in yeast vector. 1. PHRE1 5’ LTR pLacZi (320 bp), 2. TCP20 AD (640 bp) 3. GATA AD (390 bp), 4. DOF2 AD (470), 5. *PHRE2* 5’ LTR pLacZi (505 bp). **(C)** PCR amplified products for cloning in BIFC vector. 1. *PHRE1* BIFC (5005 bp), 2. TCP20 BIFC (680 bp), 3. DOF2 BIFC (530), 4. *PHRE2* BIFC (5603 bp), 5. GATA BIFC (582 bp).
