# Supplementary material for: Long terminal repeats (LTR) and transcription factors regulate PHRE1 and PHRE2 activity in Moso bamboo under heat stress
Source: BMC Plant Biol. 2021 Dec 9;21:585. doi: 10.1186/s12870-021-03339-1 (PMC8656106; doi:10.1186/s12870-021-03339-1)
Supplement: Supplementary file 2 — Additional file 2. [file 12870_2021_3339_MOESM2_ESM.docx]

**Supplementary Table 1. Long terminal repeats (LTR) and transcription factors regulate *PHRE1* and *PHRE2* activity in Moso bamboo under heat stress**

>input213370_RT16_B3_L61_390 -PHRE1 LTR retrotransposon

GAGAACTCATGTCATGGGCATGTCAGCACTATGAAGTAACTGACGAAGAGTTTAGGGTAATTGAGAAAGTATATGTTAAGCAAGTAGTCAAGCATCAGAATTCAGAACCTTAATGGGCTGCAAGCAGTTATTCGGCCCATAGGTGTGGGTGTGAGTGTAACGCCAATTGTCGTATTCGCTACAGCCTTCCGGCTTGTAAAGGGCGTGAAGGGGTTGGGGAAGGGCATCGAACAAGAACCTAACTGCTTTCGCAACTATTTTCCTCTACTGTTCTTGCTAAACCTTCCGATTTCTGAGTCTATCGGTGCTTGATTGTTTCGATTCTTTGGCGTGACGCCGGGAACGTTACATATTGGTATCAGAGCTAGCGACGCAGGGCAATCTGGCGAGAAAGAAGGGATCGCAACCCACTCTGACGGCAAGAATGGAGGCGGCGGAAGGGAGAGTATCTTCTCTGGATGACGACGTCAAAGCGATCTTGATGATGGTCACGTCGATGGACGACCGCATGAAGCAAGCGGAAGCCTGGAGGCCTACGGTTGATTACACACTGACGGGGCTGCGGCAAGTTACAGAAGATCTCAAACTGCGGGTGTCCAATCTGGAAGGAGTGCCGCCTGGGCCATCCCGTCCAGCGACGCCACGGGCTGAAGGTCATCGAGAAGAAAGACAACACTTGAGCGATGCTCATGGAGTTTTCGTCAATTCTGGGCAAGCCCCGACTAGAGGTGAGATCTCATACCAGAAACCCCAGACTGCTTATGAGTCGGGAGCAGGATTTGGTAGGGGAATGTGTGGTACTCCAACAAATTTCAACTGTCAGCACAGGGAGTTCCGCATGCCTAAAGCTGAATTTCCAAGATTCAATGGGGAGCATCCCAGGTTATGGAAAGATAAGTGCGAGAAATTTTTTCAGATGTTTCATGTTCCGATGCAGTTTTGGGCTCAATATGCCTCCTTGCACTTCCAAGGCAATGCGGCATTATGGTTGCAAACATTTGAAGCTCAGCATTCTGCGGAAGATTGGGTGGAGCTATGCATTGCCATAGACGCCAAATTTGGGAAGGATCTATATCACAATTACATGAAGACACTATTGAGTATTCGTCAATTGGGAGAAGTTCAAGAATACTATGAGAGATTCCAAAATGCTGTGCATAAAGTGCTAGCACACAATAACAATTATGATGATGTGTTTTTTGTTAACAAATTCATAGATGGTCTGAAACCAGACAATGCTCTGAATCTCATTTATTCGACCGAACAGTTAGCTCCATTGTCCATACTGGCAACTCGACTCAGCAAAGTTGGACGCAAGGTGATTGACCAAGGCTTAATTCAATGGTCCCAAGCACCGGGGAGATTAGGTCAGCACTGGTATTGCACAAGCCTCGGACTGTAGATGCAGCACTGTCCTTAGCACTATTACAAGAAGAAGAATTGTCAACCTCGGCAAGGAAGTATCATGGACGTTCTGAAAACAGAGACTACACCAAATTCAAGTCTCAAGTATATGGAGGAAAAGGCATTCTGGGAACTGCTCCGACTGAACCCAGGCTGGACAACAAGCTGAAAGTCGATGAAAAGTATGAAAGTTTGCGATCATTACGCAAGGCTCAAGGACTATGTATGAAATGTGGTGAAAAATGGGGAGTGGGACATAGGTGCCCAAAGCAAGTCTCATTACATGTGTTGGAAGAACTGCTGGAAATAATGCAATTAGAAGGAGAGAATACTGAAGATATGGATGCTGACAGTAAAAGTGGTAATGAGGAAGTATTGATGAGCATATCACATTCTGCAGCTGTGGGGATTCAGGGCAAACGAACCATCAGATTGCAAGGCATTGTTAGAGACCATGAAGTATTGATATTGGTTGACTCAGGCAGTTCTGGAAATTTTGTCAGTGAGGCCTTAGTGCAGCGATTGCAATACCCGACGAAGAAGACAAATCCAGTCCAGATCTCCTTAGCCGATGGCAAAAGAGTATACAGTGACCAGCAAATTGCAAGTCTCACTTGGTGGACTCAAGGATACACTTTTGCAACAAATGTCAGGGTGCTCCATTTGGGATGCTATGACATGATTTTGGGTATGGAATGGTTAGAAGAACAAGGTCCTATGTGGATAGACTGGCAAAGGAAAAAACTGAGATTCATGCATCTAGGCAACAGGATTACCTTGTATGGTGTAAAGGATGTCACTAACACATGCCAGGAAATTTCTGCAAAAAGGCTCCACAGTGTGGTCCGACAGGGAGGTATTGCTCAACTTGTGCAATTGACTGCAGTAGAAGAGCAAAATCATTCAGAAGTGATACCGCCAGCAGTGCAAACAATATTACAACAACATGCTGCCTTATTTCAAGAACCAAGTGGACTACCTCCACACAGACCGTTCGATCACTCTATTCCATTACTTCCGGGAGTTAAACCGGTGAACATTCGACCGTATAGGTATGCACCAAAACAAAAAGATGAAATTGAGAAACAGATAAGAGAAATGTTGAATCAGGGTGTCATCCAACCAAGTTCTAGCCCCTTTGCTTCTCCAGTGCTATTGGTGAAGAAAAAAGATGGCACATGGCGTTTTTGCGTGGACTATAGGCAGCTGAATGCATCCACAGTTAAAAACAAGTACCCATTACCTGTCATTGACGAACTTCTCGATGAATTACATGGTGCAAAGTGGTTCACCAAGTTGGACTTGAGGGCAGGCTATCACCAAATCAGATTAGTAGAGGAGGATGAATATAAAACAGCATTTAAGACCCACAATGGCCATTGGGAGTTTCGGGTAATGCCATTCGGTCTTACAAACGCACCGGCTACGTTTCAAGCAGTGATGAACACTATCTTTTCCGAGTTGCTTAGACAGTGTGTGCTGGTTTTTGTTGATGACATACTAGTTTATAGCAAAACTTTGGATGAACACTTGCATCACCTGCAACAAGTATTCACAATCTTAATGGAGAATCATCTATTGATAAAACAGTCCAAGTGTTCCTTTGCCCAGCAGAAACTTGAATACTTGGGACACATAATCAGTGATCAAGGTGTCGCCACTGATCCTTCAAAAATTCAGGCGGTCCAACACTGGTTACCTCCCAAAAATGTCAAACAACTCCGAGGGTTCTTGGGTTTGTCAGGTTATTACAGGAAATTCATCCGGAATTATGGTGTGATTAGCAAAACATTGACCGAACTATTAAAGAAACATACAGTGTACAATTGGACAGCTAGGGAACAAGCTGCCTTCGAAGCTATACAGCAAGCATTGGTGCAAGCACCAGTATTGGCGTTACATGATTTCACTAAGCCATTTGTTTTGGAAACCGACGCTAGTGATGTGGGCATTGGAGCCGTACTCATGCAACAAGGGCACCCGATCGCTTATTTAAGTAAGGCATTGGGGTTTAAAGCAAAAGGTTTATCTACATATGAGAAAGAGTGCTTGGCCATACTAATGGCCATTGATAGGTGGAAACCGTATCTACAACATGCAGAATTTACAATTGCAACCGACCACAAGAGCCTTTTGCACCTGGGGGAACAGAGACTTAACCAAGGAATGCAGCACAAAGCATTTCTCAAGTTACTTGGTTTACAATACAATATCATATATAAGAAGGGTCTCGAAAATAAAGCAGCCGATGCACTATCTCGCAAAGACCATCCGGTTACATGCTCAGCAATATCTGTTAGTCGTCCGAGATGGCTGGAGATTGTGGTTGAAGGTTATCAGAAAGATCCAGATACAAAAGCACTATTAACTCAGTTGGCTCTTACTGGATCTAATGCAGAGGGGTTTCTCTTGCAGGAAGGCATTATCAAATACAAAGGAAGAGTTTATTTAGGCAAACACACCGCAGCACATCAAGCAGTGTTAATGGCATTACATGACAGTGGAGTGGGTGGTCACAGCGGTATTACTGCTACATATCACAAAATTAAAGCCTTATTCGCATGGCCTGGCATGAAGAAAGATGTTCACAAATTTGTGTCAACCTGTACCGTTTGCCAACAAGCCAAATCTGAGCACATCAAACAGCCAGGACTATTACATCCACTGCCGATTCCATCCCAAGCATGGACCACTATCAGCCTCGACTTCATTGAAGGACTGCCCAAGTCTAAACAATTTGACACCATTGTGGTAGTAATCGATAAATTTTCCAAGTATGGTCGTTTTATACCCTTATCTCACCCATTCACAGCTTGGACAGTAGCACAATTATTCATCAATAACATTTACAAGTTGCATGGACTACCACAAGTCATCATCTCCGATAGGGACAAAGTCTTCACGAGCACCTTATGGCAGGAATTGTTCAAATTAACAGATACCAAGTTGAACATGAGCTCTTCTTATCACCCTCAAACGGATGGTCAGACGGAGAGGTTGAATCAATGTCTTGAAACATATCTCCGATGCATGGTACAAGCATGTCCAAACAAGTGGTCTCAATGGCTAGCACAAGCAGAATTTTGGTACAATACAACCTATCATTCGGCATTGGGTAAAACACCTTTCGAAGTGTTGTATGGCCATCCACCAAGACATTTCGGTATTCTTACTGGAGATGGCAGCTCTGCACCAGAGCTAGAACAGTGGCTGCAAGAACGTGCTGTAATGATTAAATTGATTCAACAACACCTGCTGCGAGCTCAGCAAAGAATGAAGGATCAGGCTGATAAGAAACGCTCAATTCGGGAATTTCAAGTGGGAGATTTAGTCTATCTCAAGCTCCAACCATACACTCAGCTTTCTGTCGCCCGGAGGTCCTCTCAAAAGCTGTCCTTCAAATACTTTGGCCCTTACAAGATTATACAGAAAATAGGCAAAATGGCATACAAATTGGAATTGCCGGCAGGCAGTCAAATCCATTCAGTTATACATGTATCTCAACTGAAGAGAGCCATCAAACCAACAGAAACAGTCAGCAGCACATTGGTCCACATGGGAGAGTCTGGCAGACCTACGCAACCGATTTCCATTAGCGCCAGCTTGGGGACAAGCTGGTCCTTAACAAGCTGGTCCTTAAGTGGGGGAGAATGTCAGCACTATGAAGTAACTGACGAAGAGTTTAGGGTAATTGAGAAAGTATATGTTAAGCAAGTAGTCAAGCATCAGAATTCAGAACCTTAATGGGCTGCAAGCAGTTATTCGGCCCATAGGTGTGGGTGTGAGTGTAACGCGAATTGTTGTATTCGCTACAGCCTTCCGGCTTGTAAAGGGCGTGAAGGGGTTGGGGAAGGGCATCGAACAAGAACCTGACTGCTTTCCCAACTATTTTCCTCTACTGTTCTTGCTAAACCTTCCGATTTCTGAGTCTATCGGTGCTTGATTGTTTCGATTCTTTGGCGTGACGCCGGGAACGTTACAGGG**CATACTTAATCTGACACCAGA**

Red colors indicate 5’LTR and 3’ LTR sequences

Maroon color indicates gag sequence

Blue color indicates pol sequence

POLYPURINE TRACT (PPT): CAAGCTGGTCCTTAAGTGGGGGAGAA

Primer binding sites (PBS): TATTGGTATCAGAGCTAGCGACGCAG

**>in****put220000_RT6_B3_L15_984 -PHRE2 LTR retrotransposon**

TGTCAGGACTGGATTATTCAGAGAAGCTGAAGTCGCTGACAGAGAAGAACTGAAGACCACCAGACTTGACTGAACTTATGAGTTCTGTCAAGTCGGCAGTCTCCTTACCGATTCGTCGTTTTCACTTGAACCAAGATGGAGGGCTAAGATGTGCACGTAACTATCGGGTGGAGGATCCAACGATCAACAGCAGACCGCGTATTTACTGTTTTGCGTCATCTCCCTTGTCTTGTAACTGAACTTTTGAACTCAGTCATTTTTATTCCCAGCTGTAAGTTTGTTCGGATTTGTCCCTGGCTATATAACCAGCGGAAGTGAAAGGAGCAAGGCATCGAACAGCATATACCTCTACTTTAGTTTGTTAGATTGTAGAACCCTAACTCGTTTTCCTCTTTTTCTCACCGTTTCACCCGCACAGACCGCAAGTTCAATCCCTGAACTTATGAGTTCTGTCATTTGGTATCAGAGCAACAGCAGATCCTGATCCGTGATTTGCAAGAGCGAAGGTTGGGGAAGTCGCAAGATTTTCCGCCGTAAAATCCCTTAGCTTAAGCAGATCTGGGAGTGGATGGTGGCAAAGACTAGATCCCACGGATCGATTGAGGAGGAAGGTATTGAGATGGAAAACCGGGCTGAAATCCTCAAGATACAGGAGAAGTTGTTGGAGCTTGATGCCCTGAGGTCAGATGTGGCCGAAATCAAAAATCTTTTATTTACAAAATTTGCCACACCTTCATCGGCTGGGGCGGAAGTGGTGGTCACTGAAGCTAGAACCGAAGGACCAATACCGACTGAAGTCTGGCCGAATCAGGCATGGAATGCAGAGGGGAACCGGAGGACTAGAGCGCACCCTGCTTTCTTCGTGGAGCCGAACATGGTAATACCAAACCAACCCCCGATTTACTCGCCTGCAATTAGAACCACTTCACAAGTAGAGTTGGGGGAAGGATCTGGAGAACATCACCGGGGATTATGGCAGACATCTGTAGCGCTGCCAGAAAGGTTTCAGCAAAGAGGAAACTATGGTCCTGGGGTGCCAGCAGCACCACTTTGGTCAGGACCGATTACTAACCCAGAAATGTTCCCTCAAGGAGTAAACCGTGACTCGGTATTACCAGCAGCACCCGCCTGGTCAGGACAGAACCCCAACCCGGCAGCAGGTATGCAGTCATATTACCACCCGCCTTGGAATATTGCTCCAGGTAATGATAATCAGTTGCCACACAACAGTTGGGCTTATACTTCAAACAACCCAGGAGTGAATTTAGTTTATCAACTGGACAACCAGAATCCAAACATGAACAGTAGAGGGCATCAATATGCAGATGCGGTGATTAGGGGCCCGAAGCTGGAAATACCTTTGTTCACAGGTGAGGATCCAATCGGGTGGCTCGAGCAATGTGGAAATTTTTTTGAAATGGCTGGGACACCCAGAGAACAGTGGGTTAATATTGCTTCGGGACATTTCACCGGAAGAGCTCACACTTGGTTTAAAGGGATTGGAATTGCTTGGCAAGCACTTAATTGGCAGCAACTTTGTGCAATGATAGCTGACAGATTCGCTGAGGCCAGTGCTCATGAGGCGGTGGAAAGACTTCAAACAATGAAGCACATGGGAAATGTTGGCAAATATATTGATTACTTTGAAAAAAGTGTGGAGCTAGTGAGAAGGGACCATCCATATCTACAAGAAGCCTTTTTATTGAGTTGCTTTATTGGTGGTTTGAAGGATGAGATCAAGTTTGGAGTGAGTATGCACCAGCCAAAGGGACTGCTTGAGGCATATTGGTATGCTAAATTAGAAGAGAAGGCAGTCTTAGCTAGAAGGTCTGGGTCATCTGGAGGTTATAACAGGAACAGACCAGCTAACAATCCACTCAGGCAAGGACCAGTTAAAGGAAACATGGTAGAAACCAATGAGAGGGAAAGAACTCCAAAACAAGAGGGGAACAAGAGGACCTGTTGGCATTGTAATGAACCCTGGGTTCCTGGCCATAACTTGAAATGTAAAATCAAGAAAGCCCTACATGCTATTTTGATGCAAGGTGATGAGGAGGGAGAAGAAAATACTGAAGACATGGAAGTAACCAAAGACCATGGGTTTATAACAGCTCCTGGAAGCCCTGAAGAGCTAGTAGAGGCTCAAGTGAATGCACAACTATTGGTAATCTCAAGTCAAGCCATAGCAGGAGGTACAGGCTCTGCCACGTTTACTTTGAGAACCAGAATTGGAGGAAAACCGGCAGTAATACTTGTTGACAGTGGCAGCACTCACTCTTTTATGGATTATGAGTTTGCAGTCAAAACTGAATGCAAGTTTTTATATCAACCGATCAAGAAAATAGCAGTTGCTGGAGGTGGAGAACTATGTTCAGATGCAAAAACTGAGAAGCTACCTTATGTGATTCAAGGCTGTCATTTTATCTCTTCATTTCAATTACTTAAGCTCAAAAGCTATTCCATTATCCTTGGGGCTGATTGGATCTATGAACATAGTCCGATTGGGTTAGATCTTAAGACCCGGGAATTGACTATCTGTAAGGAGGGACAAAAGGTGACTTTCAAAGATTATACACTCCCTGATAAACATTGTCTAGTAGGAGCGACTCGGTTAGAGAAGATGATGAGAAAAGAGGTGTTGGGCTGCATGATTCAAATAAATTGGGCAAAAGAAGAAACTGAACTGGTGACAGAAGAGTTACCTACAGAAGTGCAGAAACTATTGCAGCAATACTCTGATCTGTTCAAGGAACCGAAGGGCTTACCTCCAAAGAGAGAATGGGACCATAGCATTCCACTTAAAGGCGATGCAGATCCACCAAACTTAAGGCCTTATAGAACTCCGCGTCACCAAAGGGAAGCCATGGAAAATATTATCAAGGAATTATTAGAGTCGGAAGAAATCAAGGTTAGTGTGAGTCCTTATTCCTCCCCGGCCATTATGGTTAGAAAAAAAGATGGATCTTGGAGATTGTGTGTGGATTATAGGCAACTTAATTCAATGACAATCAAAAACAAATATCCGATGCCCATCATAGAAGATCTCTTGGATGAGTTACATGGGGCGACTATTTTCTCTAAGTTGGACCTTCGGTCCGGCTATCACCAAATCAGAATGGCAGAGGATGATATCTCAAAGACTGCTTTTAGAACTCATTCAGGTCACTATGAGTATACAGTTATGCCTTTTGGGCTAACGAATGCCCCAGCAACTTTTCAATTCCTTATGAACCACATATTCCATGAGCAGTTGAGGAAGTCCGTACTTGTATTCTTTGATGACATTCTCGTCTTTAGTAAGAATCTGTCAGACCATGTGCAACATTTACATCAAGCATTTGAAATACTGAGACAAAATCAATTGTTTGTGAGAAGAACCAAATGCAGTTTTGCTGTAGCCGAAATTGGATATTTGGGCCATATTATCAGTGGTGCTGGAGTGGCAACAGATCCAAGAAAGATAGTCGATGTGGTGAATTGGCCTACACCAAATTCAGTCACCAAATTGAGGGGATTTTTGGGCTTAACCGGATATTATAGAAGATTTGTGAGAGGGTATGGAAAGGTATGCCAACCACTCTTTGACATCTTAAAGAAGGACTCCTTTAATTGGGGTGAAGCACAAACAATAGCTTTTGAAACTCTAAAAAGGGCTATGACTACCTGTCCAGTTTTAGCTTTACCGGATTTTACTCAACCTTTCATCCTAGAGACGGATGCTTGTGGTACTGGACTTGGGGCTGTTCTTATGCAAGGTAAAAGACCTATTGCTTATTATAGTAAAACCTTGGGTGCAAGGGCAGCAACACAATCTATATATGAAAAAGAAGCAATGGCAATTCTGGAGGCTTTGAAGAAATGGAGACATTATTTATTGGGGAATCAGCTGCTGATCCGAACTGACCAAAAAAGTCTGAAGTTCATCACTACCCAAAGAGTCTCGGAGGGAATCCAACACAAATTACTTCTCAAGTTACTAGAATTTGATTACAAAGTGGAGTATAAGAAGGGAAAAGAGAATCTGGCTGCAGATGCATTATCAAGAAGAGATGTTAACCCTCAAGAGAGTGAAGAAAGATGTCATGCTATAGTTACTATTATGCCCGAATGGGTGGAGGATGTGAAAGGAAGTTATGTCAATGATCCTCAATATGTGAAAATGGTAACAAATGATCATCTTCAAACTGGCATTGACAGTAATTTTACCTTGGAGTCAGGATTAGTAAGGTATAAAGGGAGAATTTATGTGGGGGTGGGAAATGACAGCCGAACTAAGATTATGGACTCTTTTCATTCATCATCCATTGGAGGGCATTCTGGAAGGCGAGCAACCTATCACAGAATTAAAAAGTTATTTTATTGGCCTAAACTCAAGAGAAATGTGGAAGCATTAGTTGCTGAGTGTCCGGTCTGTCAGATTACCAAGTCAGAACATATTCATATCCCTGGGTTGCTAAACCCTTTAGCAATACCGGACATGGCATGGACACATATAAGCATGGATTTCATTGAGGGATTACCTAAATCCCGAGGCAAGGAGGTAATTTTGGTGGTAGTGGACCGGTTAACTAAATATGCTCATTTCTTGTCCCTAGCTCACCCATATTCAGTTCAACATGTGGTACAAGCTTTCATGGACAACATTTTCAAATTGCATGGGATGCCCATTGCCATAGTGACTGACCGCGACAGAATATTCACTAGTCACTTGTTTCAGGAGATCTTTAAGATGTTGAAGGTATCTTTGAGATTGAGTAGCGCTTATCATCCACAAACGGATGGACAGACAGAAAGAGTGAATCAATGCTTGGAAGCATATTTGAGGAGTATGACTTTTCAAGAACCTAGAGAGTGGATGAATTGGCTCACACTTGCCGAGTGGTGGTATAACACAACCTACCATACTTCATTGAAAGTCACACCATTTCAAGCTCTATATGG

ATATCCACCTCCTCTAGTGGGAGAACTTTCTATCCCTTGCAATGTGTCAGAAGAAGCAAGAATCATTGTTGAACAAAAGGAGCAAATGTTAGAGCAGCTGAAATTCAATCTGCACAATACCCAGGAGAGAATGAAGCATTATGCTGATAAAAACCGAACCGAAAGGCAATTCCAAGTGGGTGACATGGTCTATCTCAAAATGCAACCTTATAGGCAAGCTGCTTTTGGTATAAGGGGGTCTCTTAAGCTTCAGTCTAAATATTATGGTCCATTTCGAGTGCTGGAAAAACTGGGAGGGGTGGCTTACCGATTGCAACTTCCAGAGCATACTTTGATCCATCCCGTTTTTCACGTGAGTCAACTAAAGAAGCACTTGGGACCACAGGCAGTACCTCTTCCTAGACTACCGTTGGTTGGTGAAAATGGTAAAATCAAAACTGAGCCCATAGCTGTTCTGGACCGGCGCATTGTCCCTCGCCGAAATGAGCCGGTTGCGCAGCTGTTGATCCAATGGTTGAATCTCGGTCCTGAAGATGCTACCTGGGAAGACTTGTCGTTCATCCGGAAGACCTTCCCG**AGTTTTACTCCTTGAGGA**CAAGTCGATTTCCAAAAGGGGGGAAT**TGTCAGGACTGGATTATTCAGAGAAGCTGA**AGTCGCTGACAGAGAAGAACTGAAGACCTCCAGACTTGAAGTCAGCAGTCTCCTTACCGATTCGTCGTTTTCACTTGAACCAAGATAGAGGGCTAAGATGTGCACGTAACTATCGGGTGGAGGATCCAACGATCAACAGCAGACCGCGTATTTACTGTTTTGCGTCATCTCCCTTGTCTTGTAACTGAACTTTTGAACTCACAGTCATTTTTATTCCCAGCTGTAAGTTTGTTCGGATTTGTCCCTGGCTATATAACCAGCGGAAGTGAAAGGAGCAAGGCATCGAACAGCATATACCTCTAGTTTAGTTTGTTAGATTGTAGAACCCTAACTCGTTTTCCTCTTTTTCTCACCGTTTCACTCGCACAGACCGCAAGTTCAATCCCTGAACTTATGAGTTCTGTCA

Red colors indicate 5’LTR and 3’ LTR sequences

Maroon color indicates gag sequence

Blue color indicates pol sequence

POLYPURINE TRACT (PPT): CAAGTCGATTTCCAAAAGGGGGGAAT

Primer binding sites (PBS): TTTGGTATCAGAGCAACAGCAGATCC
