# Supplementary material for: Long terminal repeats (LTR) and transcription factors regulate PHRE1 and PHRE2 activity in Moso bamboo under heat stress
Source: BMC Plant Biol. 2021 Dec 9;21:585. doi: 10.1186/s12870-021-03339-1 (PMC8656106; doi:10.1186/s12870-021-03339-1)
Supplement: Supplementary file 4 — Additional file 4. [file 12870_2021_3339_MOESM4_ESM.docx]

**Supplementary Table 3**. List of primers used for used in this study. The annealing temperature for PCR and RT-qPCR was an experiment at 60-65°C.

| **Purpose** | **Primers ID** | **Forward primer (5'-3')** | **Reverse primer (5'-3')** |
| --- | --- | --- | --- |
| LTR s and TFs isolation | PHRE1 | GAGAACTCATGTCATGGGCATG | TTAAGGACCAGCTTGTCCCCA |
|  | PHRE2 | TGTCAGGACTGGATTATTCAGAGAA | TCCTCAAGGAGTAAAACTCGGG |
|  | TCP20 | GAACCTGAACAGGCACCAG | TCCTTCTCGCCGCTCTC |
|  | DOF2 | TGGCCAAGGAGGAGAGG | GGGCAGGTTCAGGAACAG |
|  | GATA | ATGCTAGATCCCACCGAGAAAGTA | TTAAGCATAAACGGAAGAAG |
| Transformation | PHRE1 5’+ gag+pol LTR | TTTCTCAAGATCAGAAGTACTTGTCAGCACTATGAAGTAACTGACGA | TTAAGGACCAGCTTGTCCCCA |
|  | PHRE1 GUS | CAGCTTGGGGACTAGATCATTGTATCTACTGTGGCGC | GACCAGCTTGTCTAATCGGAAAAGTTAAAGTCTTTCTT |
|  | PHRE1 3’LTR | TCCGATTAGACAAGCTGGTCCTTAAGTGGGG | TCTGGTGTCAGATTAAGTATGCCCT |
|  | Hygromycin (hptII) | CGAAATTGCCGTCAACCAAGCTCT | CATGGCGTGATTTCATATGCGCGA |
|  | PHRE2 5’+ gag+pol LTR | TCAAGATCAGAAGTACTTGTCAGGACTGGATTATTCAGAGAA | TCCTCAAGGAGTAAAACTCGGG |
|  | PHRE2 GUS | CTCCTTGAGGACTAGATCATTGTATCTACTGTGGCGC | CCCTTTTGGAAATCGACTTGTCTAATCGGAAAAGTTAAAGTCTTTCTT |
|  | PHRE2 3’LTR | CAAGTCGATTTCCAAAAGGGG | AAAACGACGGCCAGTGAATTCTGACAGAACTCATAAGTTCAGGGATT |
|  | GW PHRE1 | GGGGACAAGTTTGTACAAAAAAGCAGGCTTGTCAGCACTATGAAGTAACTGACG | GGGGACCACTTTGTACAAGAAAGCTGGGTTTCTGGTGTCAGATTAAGTATGCCCT |
|  | GW PHRE2 | GGGGACAAGTTTGTACAAAAAAGCAGGCTTGTCAGGACTGGATTATTCAGAGAA | GGGGACCACTTTGTACAAGAAAGCTGGGTTACAGAACTCATAAGTTCAGGGATT |
| qRT-PCR analysis | PHRE1 5’LTR | GAACTCATGTCATGGGCATGTC | ATGGGCCGAATAACTGCTTG |
|  | PHRE1 gag | CCAAGATTCAATGGGGAGCATC | AACCATAATGCCGCATTGCC |
|  | PHRE1 pol | TTCAGGGCAAACGAACCATC | TTCGTCGGGTATTGCAATCG |
|  | PHRE1 3’LTR | AGCAAGTAGTCAAGCATCAGA | GTCACGCCAAAGAATCGAAAC |
|  | PHRE2 5’LTR | TTCTGTCAAGTCGGCAGTCTC | ACAGTAAATACGCGGTCTGC |
|  | PHRE2 gag | GCTAGAACCGAAGGACCAATAC | GTTCGGCTCCACGAAGAAA |
|  | PHRE2 pol | ACTTGGGGCTGTTCTTATGC | AGCCTCCAGAATTGCCATTG |
|  | PHRE2 3’LTR | TGCGTCATCTCCCTTGTCTTG | TTCGATGCCTTGCTCCTTTC |
|  | Actin (*Arabidopsis*) | CTCAGCACCTTCCAACAGAT | CACCACCACGAACCAGATAA |
|  | NTB (Moso bamboo) | TCTTGTTTGACACCGAAGAGGAG | AATAGCTGTCCCTGGAGGAGTTT |
|  | TCP20 | CCACCGAGATCAAGGACTTC | CCTTGTTGCTGCTCCTCTT |
|  | DOF2 | GGACACCAAGTTCTGCTACTAC | TGCTGCTGCTGCTCTTC |
|  | GATA | CTAGATCCCACCGAGAAAGTAATC | CCACAAATGGCACAACTCTTC |
| Insitu Hybridization | PHRE1 5’LTR | CACTATGAAGTAACTGACGAAGAGTT | ACGCCAAAGAATCGAAACAATCA |
|  | PHRE2 5’LTR | CCACCAGACTTGACTGAACTTAT | TCGATGCCTTGCTCCTTTC |
| Yeast one hybridization | pLACZi 5’ LTR PHRE1 | CTTGAATTCGAGCTCGGTACCTGTCAGCACTATGAAGTAACTGACGA | ATACAGAGCACATGCCTCGAGCGGCGTCACGCCAAAGAATCGA |
|  | pLACZi 5’ LTR PHRE2 | CTTGAATTCGAGCTCGGTACCTGTCAGGACTGGATTATTCAGAGAA | ATACAGAGCACATGCCTCGAGGATACCAAATGACAGAACTC |
|  | TCP20 AD | GAGTGGCCATTATGGCCCGGGAGGAGCCCAACAAGAAGAGC | GCCGACATGTTTTTTCCCGGGAAGCTCTGGGGGTTCAGCAC |
|  | DOF2 AD | GAGTGGCCATTATGGCCCGGGTGGCCAAGGAGGAGAGG | GCCGACATGTTTTTTCCCGGGGCCGCCGCTGCCGGCGTCG |
|  | GATA AD | GAGTGGCCATTATGGCCCGGGCACAGCAGCAGTAGCAGTAA | GCCGACATGTTTTTTCCCGGG TTAAGCATAAACGGAAGAAG |
| Subcellular localization | BIFC PHRE1 | GGCCTGGCGCGCCACTAGTTGTCAGCACTATGAAGTAACTGACG | CCTCGAGGTCGACAGTACTATCGATTTAAGGACCAGCTTGTCCCCA |
|  | BIFC PHRE2 | GGCCTGGCGCGCCACTAGTTGTCAGGACTGGATTATTCAGAGAA | CCTCGAGGTCGACAGTACTATCGATTCCTCAAGGAGTAAAACTCGGG |
|  | BIFC TCP20 | GGCCTGGCGCGCCACTAGTAGGAGCCCAACAAGAAGAGC | CCTCGAGGTCGACAGTACTATCG GGTGAAGCTCTGGGGGTTC |
|  | BIFC DOF2 | GGCCTGGCGCGCCACTAGTGGGACACCAAGTTCTGCTACT | CCTCGAGGTCGACAGTACTATCGATGCCGCCGCTGCCGGCGTCG |
|  | BIFC GATA | GGCCTGGCGCGCCACTAGTCACAGATGCTAGATCCCACCGAGAAAGTA | CCTCGAGGTCGACAGTACTATCGTTAAGCATAAACGGAAGAAG |
