# Supplementary material for: Long terminal repeats (LTR) and transcription factors regulate PHRE1 and PHRE2 activity in Moso bamboo under heat stress
Source: BMC Plant Biol. 2021 Dec 9;21:585. doi: 10.1186/s12870-021-03339-1 (PMC8656106; doi:10.1186/s12870-021-03339-1)
Supplement: Supplementary file 5 — Additional file 5. [file 12870_2021_3339_MOESM5_ESM.docx]

**Supplementary Table 4. List of primers used for sequencing PHRE1 and PHRE2 by Sanger method**

| **S.no** | **Primers ID** | Primer sequence (5'-3') | Product size (bp) |
| --- | --- | --- | --- |
| 1 | PHRE15’ LTR1 F | ACTGATAGTGACCTGTTCGTTG | 221 |
|  | PHRE1 5’ LTR1 R | CACACCTATGGGCCGAATAA |  |
| 2 | PHRE1 5’ LTR2 F | TAGGTGTGGGTGTGAGTGTA | 269 |
|  | PHRE1 5’ LTR2 R | GGTTGCGATCCCTTCTTTCT |  |
| 3 | PHRE1 5’ LTR3 F | GGAAGATTGGGTGGAGCTATG | 251 |
|  | PHRE1 5’ LTR3 R | TCCGAGGCTTGTGCAATAC |  |
| 4 | PHRE1 5’ LTR4 F | GCCTACGGTTGATTACACACT | 508 |
|  | PHRE1 5’ LTR4 R | GCATAGCTCCACCCAATCTT |  |
| 5 | PHRE1 5’ LTR5 F | GGCACCCGATCGCTTATTTA | 931 |
|  | PHRE1 5’ LTR5 R | CTCGTGAAGACTTTGTCCCTATC |  |
| 6 | PHRE1GUS1 F | CGGTACGTGTGACTATGAGAAG | 528 |
|  | PHRE1GUS1 R | GACAGCCGAAATTGGAGAGA |  |
| 7 | PHRE1 GUS2 F | AGGATCAAACGCGCGATATAAA | 642 |
|  | PHRE1 GUS2 R | CGACACGGACTTGGCAATAA |  |
| 8 | PHRE1 GUS4 F | GAACGCTTCCTATCACCCTAAC | 753 |
|  | PHRE1 GUS4 R | GTGTCTACCAATCTCTCCGAATG |  |
| 9 | PHRE1 3’LTR1 F | GGGAGAATGTCAGCACTATGAA | 298 |
|  | PHRE1 3’LTR1 R | CAAGCACCGATAGACTCAGAAA |  |
| 10 | PHRE1 3’LTR2 F | AGCAAGTAGTCAAGCATCAGAA | 257 |
|  | PHRE1 3’LTR2 R | GTCACGCCAAAGAATCGAAAC |  |
| 11 | PHRE2 5' LTR1 F | GCCTTGTCAGGACTGGATTATT | 464 |
|  | PHRE2 5' LTR1 R | GGATCTGCTGTTGCTCTGATAC |  |
| 12 | PHRE2 5' LTR2 F | GAAGGACCAATACCGACTGAAG | 486 |
|  | PHRE2 5' LTR2 R | CACTCCTGGGTTGTTTGAAGTA |  |
| 13 | PHRE2 5' LTR3 F | TCAGGCAAGGACCAGTTAAAG | 906 |
|  | PHRE2 5' LTR3 R | CCCATTCTCTCTTTGGAGGTAAG |  |
| 14 | PHRE2 5' LTR4 F | GGAAATGACAGCCGAACTAAGA | 346 |
|  | PHRE2 5' LTR4 R | GGTGAGCTAGGGACAAGAAATG |  |
| 15 | PHRE2 GUS1 F | CGCTCACTTCTAGGGAAAGAAC | 509 |
|  | PHRE2 GUS1 R | CGCACCACTACACCTCATAAC |  |
| 16 | PHRE2 GUS2 F | TGCGCGATAGTCGAGAAATTAG | 464 |
|  | PHRE2 GUS2 R | GGCCCACTTCCAATAGAGATAC |  |
| 17 | PHRE2 GUS3 F | GAACGCTTCCTATCACCCTAAC | 753 |
|  | PHRE2 GUS3 R | GTGTCTACCAATCTCTCCGAATG |  |
| 18 | PHRE2 GUS4 F | CCTATCAGACGGTCAAGTCAAG | 640 |
|  | PHRE2 GUS4 R | GTCAAGCGGCTACGTCTATAAG |  |
| 19 | PHRE2 3'LTR1 F | GGGAATTGTCAGGACTGGATTA | 224 |
|  | PHRE2 3'LTR1 R | TCAGTTACAAGACAAGGGAGATG |  |
| 20 | PHRE2 3'LTR2 F | CAGCAGACCGCGTATTTACT | 358 |
|  | PHRE2 3'LTR2 R | ACTGATAGTGACCTGTTCGTTG |  |
